# A Novel Role of *Listeria monocytogenes* Membrane Vesicles in Inhibition of Autophagy and Cell Death

**DOI:** 10.3389/fcimb.2017.00154

**Published:** 2017-05-03

**Authors:** Svitlana Vdovikova, Morten Luhr, Paula Szalai, Lars Nygård Skalman, Monika K. Francis, Richard Lundmark, Nikolai Engedal, Jörgen Johansson, Sun N. Wai

**Affiliations:** ^1^Department of Molecular Biology, Umeå UniversityUmeå, Sweden; ^2^Laboratory for Molecular Infection Medicine Sweden, Umeå UniversityUmeå, Sweden; ^3^Umeå Centre for Microbial Research, Umeå UniversityUmeå, Sweden; ^4^Centre for Molecular Medicine Norway, Nordic EMBL Partnership, University of OsloOslo, Norway; ^5^Department of Medical Biochemistry and Biophysics, Umeå UniversityUmeå, Sweden; ^6^Department of Integrative Medical Biology, Umeå UniversityUmeå, Sweden

**Keywords:** *Listeria monocytogenes*, membrane vesicles, autophagy, listeriolysin O, pore-forming toxin

## Abstract

Bacterial membrane vesicle (MV) production has been mainly studied in Gram-negative species. In this study, we show that *Listeria monocytogenes*, a Gram-positive pathogen that causes the food-borne illness listeriosis, produces MVs both *in vitro* and *in vivo*. We found that a major virulence factor, the pore-forming hemolysin listeriolysin O (LLO), is tightly associated with the MVs, where it resides in an oxidized, inactive state. Previous studies have shown that LLO may induce cell death and autophagy. To monitor possible effects of LLO and MVs on autophagy, we performed assays for LC3 lipidation and LDH sequestration as well as analysis by confocal microscopy of HEK293 cells expressing GFP-LC3. The results revealed that MVs alone did not affect autophagy whereas they effectively abrogated autophagy induced by pure LLO or by another pore-forming toxin from *Vibrio cholerae*, VCC. Moreover, *Listeria monocytogenes* MVs significantly decreased Torin1-stimulated macroautophagy. In addition, MVs protected against necrosis of HEK293 cells caused by the lytic action of LLO. We explored the mechanisms of LLO-induced autophagy and cell death and demonstrated that the protective effect of MVs involves an inhibition of LLO-induced pore formation resulting in inhibition of autophagy and the lytic action on eukaryotic cells. Further, we determined that these MVs help bacteria to survive inside eukaryotic cells (mouse embryonic fibroblasts). Taken together, these findings suggest that intracellular release of MVs from *L. monocytogenes* may represent a bacterial strategy to survive inside host cells, by its control of LLO activity and by avoidance of destruction from the autophagy system during infection.

## Introduction

*Listeria monocytogenes* is a Gram-positive, food-borne pathogen, and the causative agent of listeriosis. The infection is especially dangerous for fetuses, newborns, the elderly, pregnant women, and immunocompromised patients, and can cause premature birth or miscarriage, meningitis, septicemia, and encephalitis. As an intracellular pathogen, *L. monocytogenes* can invade and replicate in both phagocytic and non-phagocytic cells using a variety of virulence factors (Farber and Peterkin, 1991; Vázquez-Boland et al., 2001). Upon entry, some bacteria escape the phagosome and successfully replicate in the cytosol of host cells. Phagosome escape before lysosomal fusion is mediated mainly by a key virulence factor, listeriolysin O (LLO, encoded by the *hly* gene), and facilitated by two phospholipases C (PLCs; Smith et al., 1995; Schnupf and Portnoy, 2007; Lam et al., 2012). LLO, a pore-forming toxin of the thiol-activated cholesterol-dependent cytolysins (CDCs) family, is secreted as a soluble monomer that oligomerizes upon binding to cholesterol in the eukaryotic membrane, forming pre-pore complexes that perforate the membrane creating pores (Palmer, 2001; Kayal and Charbit, 2006; Hamon et al., 2012). Pore-forming toxins (PFTs) are the largest group of toxins produced by many bacterial pathogens. PFTs are not merely unsophisticated proteins that form pores in the host membranes, but may also manipulate cellular functions in more subtle manners, as via modulation of cellular ion concentration and induction of membrane repair (Dal Peraro and van der Goot, 2016). Moreover, recent studies showed that damage of the plasma membrane by PFT can trigger autophagy (Kloft et al., 2010).

Autophagy is a conserved eukaryotic cellular mechanism for degrading and recycling dysfunctional cellular material, which accumulates upon starvation or other stress. During autophagy, a double-membrane autophagosome forms and fuses with a lysosome within the mammalian cell, resulting in degradation of the autophagosome content by lysosomal hydrolases (Shibutani and Tamotsu, 2013; Huang and Brumell, 2014). Whereas, a basal level of autophagy is necessary for maintaining cellular homeostasis, autophagy can be induced by various stress conditions such as nutrient deprivation, hypoxia, or bacterial infection. Autophagy induced by bacteria can be classified as selective autophagy (xenophagy), non-canonical autophagy and microtubule-associated protein light chain 3 (LC3)–associated phagocytosis (LAP; Kaushik and Cuervo, 2012; Lee et al., 2012). Canonical autophagy involves a cascade of events encompassing more than 30 specific “autophagy-related” proteins (Atgs) for autophagosome formation. Non-canonical autophagy does not require the entire set of core Atgs. The LAP pathway does not involve all Atgs and is mainly characterized by direct conjugation of LC3 to the phagosomal membrane (Shibutani and Yoshimori, 2014). An important step in autophagy induction is inactivation of a negative master regulator of autophagy called mammalian target of rapamycin (mTOR). The mTOR complex 1 (mTORC1) serine/threonine protein kinase activity promotes cell growth and protein synthesis by phosphorylation of downstream targets, including p70 ribosomal S6 kinase (p70S6K) and eukaryotic initiation factor 4E-binding protein 1 (4E-BP1; He and Klionsky, 2009; Laplante and Sabatini, 2009).

Pore-forming toxins (PFT) are classified as α-PFT and β-PFT, according to the secondary structure of pore-forming regions (α-helices or β-barrels; Dal Peraro and van der Goot, 2016). The majority of PFTs are β-PFTs, for example, *Vibrio cholerae* cytolysin (VCC), listeriolysin O, streptolysin O, and pneumolysin (Iacovache et al., 2008; Dal Peraro and van der Goot, 2016). Autophagy has been implicated in responses to various PFTs by two pathways. The first pathway is activated through AMP-activated protein kinase (AMPK) by inhibiting the mTORC1 in response to a drop of the cellular ATP/AMP-ratio. The second pathway is triggered by the conserved eIF2α-kinase GCN2, which promotes autophagy in response to amino acid starvation. PKR, another eIF2α-kinase, was also shown to be involved in autophagy induction upon membrane perforation. Phosphorylation of eIF2α is required for the accumulation of autophagosomes in PFT treated cells (Kloft et al., 2010; Hamon et al., 2012; von Hoven et al., 2012; Tattoli et al., 2013). It was demonstrated that the pore forming activity of LLO can induce autophagy in bone marrow derived macrophages (BMDMs; Meyer-Morse et al., 2010). Additionally, CDCs can cause lysis of the target cells or cell death via activation of apoptotic signaling, necroptosis, or pyroptosis. The fate of eukaryotic cells depends on the PFT concentration and cell type (Keyel et al., 2013; LaRocca et al., 2014; González-Juarbe et al., 2015; Khilwani and Chattopadhyay, 2015). Apoptotic cell death occurs mainly with sublytic concentration of LLO whereas a high LLO concentration can cause rapid cytolysis of the host cells (Carrero et al., 2004, 2008; Seveau, 2014).

During *L. monocytogenes* infection, a subpopulation of bacteria is targeted for degradation inside the phagolysosomes. However, some bacteria can escape autophagy by synthesizing different proteins such as ActA, InlK, PLCs, and major vault protein or by surviving inside non-acidic, non-degradative, spacious *Listeria*-containing phagosomes (SLAPs; Py et al., 2007; Yoshikawa et al., 2009; Meyer-Morse et al., 2010; Dortet et al., 2011, 2012; Lam et al., 2012). Bacterial replication within SLAPs is LLO-dependent and represents a balance between virulence factors of the pathogen and innate immunity of the infected cell. Different fates of *L. monocytogenes* within host cells were described based on LLO activity: blocking phagosome–lysosome fusion by generating membrane pores that uncouple pH and calcium gradients across the phagosome membrane; contributing to bacterial escape from the phagosome by acting together with phospholipases C; inducing autophagy of damaged phagosomes; or leading to SLAPs formation, enabling bacteria to slowly grow and replicate within SLAPs (Portnoy et al., 2002; Shaughnessy et al., 2006; Birmingham et al., 2008a,b; Lam et al., 2013). In addition, LLO alters various host signaling pathways, e.g., it can activate IκB kinase complex-NF-κB signaling to stimulate immune activity, and modulates host cell epigenetics through histone modifications and chromatin remodeling (Kayal et al., 2002; Hamon et al., 2007; Witte et al., 2012). Previous studies showed that PFTs are secreted from bacterial cells in association with bacterial membrane vesicles, e.g., *V. cholerae* cytolysin (VCC), *Staphylococcus aureus* hemolysin, *Escherichia coli* cytolysin (ClyA) and *S. suis* hemolysin (Wai et al., 2003; Thay et al., 2013b; Elluri et al., 2014; Haas and Grenier, 2015). Many Gram-negative bacteria secrete membrane vesicles (MVs) during normal growth *in vitro* and *in vivo*. MVs contribute significantly to bacterial pathogenesis, transporting enzymes, toxins and specific virulence factors to eukaryotic cells, and mediating cytotoxicity (Wai et al., 2003; Ayala et al., 2006; Balsalobre et al., 2006; Lindmark et al., 2009; Kulp and Kuehn, 2010; Jin et al., 2011; Berleman and Auer, 2013). Previously, MVs were considered a product of only Gram-negative bacteria. However, MV secretion has recently been reported in a few Gram-positive species, including *S. aureus, Bacillus anthracis, Bacillus subtilis*, and *L. monocytogenes*, despite the lack of an outer membrane (Konings and Freese, 1972; Rivera et al., 2010; Gurung et al., 2011; Lee et al., 2013). Proteomic analysis of *in vitro*-secreted *L. monocytogenes* MVs identified a plethora of proteins involved in different cellular processes, including ABC transporters, stress response proteins and cell division proteins. LLO was also one of the proteins identified by this mass-spectrometry analysis, although the role(s) of MVs and MV-associated LLO in host interactions was not investigated (Lee et al., 2013).

In this study, we analyzed MVs production by *L. monocytogenes* both *in vivo* and *in vitro* and demonstrated a biological role of MVs in pathogen-host interactions.

## Materials and methods

### Bacterial strains and cell culture conditions

The wild type *L. monocytogenes* strain EGDe and the Δ*hly* mutant were grown for MV isolation in LB (Luria-Bertani) broth with shaking for 40 h at 37°C until late stationary phase. *V. cholerae* wild type strain V:5/04, a non-O1 non-O139 clinical isolate (Swedish Institute of Infectious Diseases, Sweden, 2004), and its derivative V:5/04Δ*vcc* were grown for 16 h with shaking at 37°C for OMV isolation. HeLa cells (ATCC CCL-2) were cultured in Advanced minimum essential medium (AMEM) supplemented with 2 mM glutamine, 10% heat-inactivated fetal calf serum (FCS), 100 U/ml penicillin and 100 μg/ml streptomycin. Human embryonic kidney (HEK293-GFP-LC3) cell line (a gift from Sven Carlsson, Department of Medical Biochemistry and Biophysics, Umeå University, Umeå, Sweden), mouse embryonic fibroblasts (MEF) cell line (a gift from Michinaga Ogawa, Department of Bacteriology I, National Institute of Infectious Diseases, Tokyo, JAPAN), and RAW-Blue™ macrophage cell line (InvivoGen) were maintained in DMEM GlutaMAX™ (Gibco) supplemented with FCS, penicillin and streptomycin. Cell lines were cultivated at 37°C in a humid 5% CO_2_ atmosphere.

### Isolation and purification of membrane vesicles

OMVs from *V. cholerae* were isolated as described previously (Wai et al., 2003; Bielig et al., 2011; Elluri et al., 2014). MVs from *L. monocytogenes* were isolated as described in the earlier studies with some modifications (Wai et al., 2003; Bielig et al., 2011; Thay et al., 2013a). Briefly, culture supernatants of *L. monocytogenes* were obtained by centrifuging the bacterial cultures at 5,000 × g for 30 min at 4°C. The supernatants were filtered through 0.22 and 0.1-μm pore size vacuum filters (Stericup, Millipore) sequentially. The bacteria-free supernatants were then ultracentrifuged at 125,000 × g for 3 h at 4°C. The pellets were washed (125,000 × g, 3 h, 4°C) and resuspended in 1x PBS, pH 7.4. The pellets were subsequently used as the MV crude preparation or purified further by Optiprep density gradient centrifugation. Purification was performed using 60% Optiprep Density Gradient Medium (SIGMA) as described earlier (Elluri et al., 2014). All purified fractions were analyzed by TEM as well as by 13.5% SDS-PAGE gel-immunoblotting for LLO.

### Estimation of MV concentration

The concentration of the MVs was estimated by quantifying the protein content using the Bicinchoninic Acid (BCA) Assay kit (Thermo Scientific Pierce, Rockford, IL) or by plotting particle size vs concentration using Nanoparticle Tracking Analysis software (NanoSight).

### SDS-Page and immunoblot analysis

Bacterial whole cell lysates and infected or uninfected host cells were lysed in a sample buffer containing 200 mM DTT, 8% SDS and 5% 2-mercaptoethanol. The procedures used for SDS-PAGE and immunoblot analysis were performed as described previously (Laemmli, 1970). The following primary antibodies were used: anti-GFP (Roche, 1:5,000); anti-LC3B (#2775, CST); anti-p-mTOR (Ser2448; #2971, CST); anti-p-p70S6K (Thr389; #9206, CST); anti-4E-BP1 (#9644, CST); anti-p-4E-BP1 (Thr37/46; #2855, CST); anti-p-AMPK (Thr172; #2535, CST); anti-p-eIF2α (Ser51; #3398, CST), anti-α-actin (Sigma-Aldrich, 1:10,000); anti-LLO (Abcam, 1:4,000); and anti-VCC (1:10,000; Ou et al., 2009). Secondary antibodies, horseradish peroxidase conjugated goat anti-rabbit (Agrisera) and rabbit anti-mouse (Dako) were used at 1:20,000 dilutions. The intensity of immunoblot reaction bands were analyzed semi-quantitatively using Quantity One 1-D analysis software (Bio-Rad) or Image J.

### Labeling of membrane vesicles

MVs were stained with the fluorescent lipid dye PKH26 (MINI26 Red fluorescent cell linker kit, SIGMA), which is commonly used for a general cell membrane labeling, and the reaction was stopped by adding 1% BSA in PBS as described earlier (Rompikuntal et al., 2015). The labeled vesicles were collected after ultracentrifugation and adjusted to the initial volume.

### Hemolytic assay

Hemolytic activity of membrane vesicles and purified recombinant LLO (Diatheva) was measured using goat erythrocytes (Agrisera) at a final concentration of 10%. For this purpose, either 50 μl (containing 1.15 μg LLO) and 10 μl (containing 230 ng LLO) of crude MVs or 100 and 50 ng of purified recombinant LLO were mixed with erythrocytes suspended in 1x PBS pH 7.4 in 96-well microtiter plates, and incubated for 3 h at 37°C. LLO inside MVs was reduced with 2 mM DTT for 20 min at 25°C prior to adding to erythrocytes. Recombinant LLO, already containing 1 mM DTT, was used as a positive control. After centrifuging the suspensions, supernatants were collected and hemoglobin release was detected spectrophotometrically at OD 541 nm with a TECAN Infinite M200 plate reader.

### Transmission electron microscopy

Vesicles were negatively stained with 1% sodiumsilicotungstate (SST) and images were taken at 150,000 × magnification with a JEM1230.

### Atomic force microscopy

Washed and concentrated bacterial cultures were placed on a freshly cleaved ruby red mica (Goodfellow Cambridge Ltd, Cambridge), incubated for 5 min at room temperature and blotted dry before placement in a dessicator for at least 2 h. Images were collected within a Nanoscope V atomic force microscope (Bruker software) using ScanAsyst in air with ScanAsyst cantilevers, at a scanrate of ~0.9–1 Hz. The final images were flattened and/or planefitted in both axes using Bruker software and presented in amplitude (error) mode.

### Dissociation assay

Vesicles were incubated for 60 min on ice in the presence or absence of either NaCl (1 M), Na_2_CO_3_ (0.1 M), urea (0.8 M) or SDS (1%), as described earlier (Balsalobre et al., 2006). Samples were ultracentrifuged (125,000 × g, 3 h, 4°C) and LLO in the supernatant and pellet fractions were detected by immunoblot analysis (soluble proteins in the supernatants were precipitated with trichloroacetic acid).

### Scanning electron microscopy

Bacterial cultures (EGDe strain) were grown in 1xLB for 16 h. A drop of bacterial culture was left to set on coverslip for 1 h at 37°C in a humidified atmosphere. Subsequently, samples were fixed with 2.5% glutaraldehyde overnight at 4°C, dehydrated in series of graded ethanol, critical point dried, and coated with 5 nm iridium. The samples morphology and MVs release were analyzed by field-emission scanning electron microscopy (SEM; Carl Zeiss Merlin) using in-lens secondary electron detector at accelerating voltage of 4 kV and probe current of 120 pA.

### Cell infection experiments

For the analysis of MVs internalization, HeLa cells were incubated with 100 μg MVs, pre-stained with lipid dye PKH26, for 30 min, 1, 3, and 6 h. Further, cells were fixed with 3% PFA and blocked in 5% goat serum for 20 min (cells were washed between each step). After fixation, samples were stained in 1% goat serum with DAPI Dilactate (Molecular Probes, Life Technologies, Eugene, OR, USA), anti-CD44 antibody (ab6124, Abcam, Camridge, MA, USA), mouse anti-Lamp-1 (TM-BB6), 1:500 (a gift from Sven Carlsson, Department of Medical Biochemistry and Biophysics, Umeå University, Umeå, Sweden), mouse anti-EEA-1, 1:200 (610456, BD Transduction Laboratories), rabbit anti-LC3B, 1:200 (3868, Cell Signaling). Secondary antibodies used were Alexa Fluor 488 Goat Anti-Mouse IgG and Alexa Fluor 647 Goat anti-Rabbit IgG (Molecular Probes). Confocal images were acquired using the 60x Plan Apo VC Oil DIC N2 lens of A1 R Laser Scanning Confocal Microscope system via the NIS-Elements Microscope Imaging Software (Nikon Instruments, Melville, NY, USA). Pearson's correlation coefficient was determined using the JACop plugin (Bolte and Cordelières, 2006). The average values and standard deviation were obtained from three different images of each time point after analyzing 20–30 cells.

For thin section electron microscopy, macrophages were seeded for 16 h before the experiment at a density of 1.5 × 10^5^ cells per well without antibiotic supplement. Then, the cells were washed, incubated for 90 min (MOI = 20) with the EGDeΔ*hly* strain (exponential phase, grown in BHI media to OD = 1.0) and fixed in 2.5% glutaraldehyde in 0.1 M phosphate buffer, pH 7.4 at room temperature for 30 min. The cells were scraped off, transferred to eppendorf tubes and further fixed overnight at 4°C. After fixation cells were rinsed in 0.1 M phosphate buffer and centrifuged. The pellets were then postfixed in 2% osmium tetroxide (TAAB, Berks, England) in 0.1 M phosphate buffer, pH 7.4 at 4°C for 2 h, dehydrated in ethanol followed by acetone and embedded in LX-112 (Ladd, Burlington, Vermont, USA). Ultrathin sections (~50–60 nm) were cut by a Leica ultracut UCT/ Leica EM UC 6 (Leica, Wien, Austria). Sections were contrasted with uranyl acetate followed by lead citrate and examined in a Hitachi HT 7700 (Tokyo, Japan) at 80 kV. Digital images were taken by using a Veleta camera (Olympus Soft Imaging Solutions, GmbH, Münster, Germany).

In the case of immunogold labeling, cells were fixed in 3 % paraformaldehyde in 0.1 M phosphate buffer. Samples were then infiltrated into 2.3 M of sucrose and frozen in liquid nitrogen. Sectioning was performed at −95°C and placed on carbon-reinforced formvar-coated, 50 mesh Nickel grids. Immunolabelling procedure was performed as follows: grids were placed directly on drops of 2% BSA (Sigma fraction V) and 2% Fish gelatin (GE Healthcare, Buckinghampshire, UK) in 0.1 M phosphate buffer to block non-specific binding. Sections were then incubated with the primary anti-Listeria O antiserum type I (BD), directed against a somatic O-antigen surface protein of Listeria, diluted 1:100 in 0.1 M of phosphate buffer containing 0.1% BSA + 0.1% Gelatin overnight in a humidified chamber at room temperature. The sections were thoroughly washed in the same buffer and bound antibodies were detected with protein A-coated 10 nm gold particles (Biocell, BBInternational, Cardiff, England) at a final dilution of 1:100. Sections were rinsed in buffer, fixed in 2% glutaraldehyde, contrasted with 0.05% uranyl acetate, embedded in 1% methylcellulose and examined in a Hittachi 7700 (Tokyo, Japan) at 80 kV. Digital images were taken by a Veleta camera.

For autophagy assays, stably transfected HEK293 cells expressing GFP fused with LC3 (autophagy marker) were seeded in 24-well plates (Thermo Scientific Nunclon) with or without coverslips for 16 h (1.5 × 10^5^ cells) prior to the experiment. The next day, media was changed and vesicles from *V. cholerae* (200 μg) and *L. monocytogenes* (250 μg) were incubated together with cells for 6 h. For the autophagy assay with purified recombinant LLO or VCC, HEK293 cells were first infected with *L. monocytogenes* MVs (200 μg) for 4.5 h and then pure LLO (250 ng) or pure VCC (2.4 nM) were added for 1.5 or 5 h, respectively. For the autophagy assay with Torin1 (Tocris), MVs from the EGDe strain were first incubated with HEK293 cells for 3 h, then Torin1 50 nM was added for 1 h, and Bafilomycin A1 100 nM was added for an additional 2 h. Cells were collected for immunoblotting, confocal microscopy or LDH sequestration assay.

For autophagy analysis by microscopy, cells were fixed with 4% paraformaldehyde and permeabilized with 0.5% Triton X-100. Cell nuclei were stained with DAPI (Sigma-Aldrich), 1:5,000, and coverslips were mounted in DAKO fluorescence medium. Microscopic analysis was performed using a NIKON D-Eclipse C1 Confocal Laser with a NIKON Eclipse 90i Microscope. Images were captured with a NIKON color camera (24 bit), using a plan Apo NIKON 60x objective.

For autophagy analysis by immunoblotting, after the indicated incubation time, cells were collected and centrifuged. The pellet was lysed and LC3 lipidation was detected by immunoblot analysis using GFP-antiserum or anti-LC3 antibody (for endogenous LC3). The stripped membrane was re-probed with α-actin antiserum as an internal control. Changes in LC3-II levels were calculated as LC3-II to actin ratio.

In a bacterial protection assay, wild type MEFs were incubated with MVs or 1x PBS for 5 h, and infected with the wild type EGDe strain (O.D = 1.0, MOI = 10), as described previously (Birmingham et al., 2007). Cells were washed after 1 h or 45 min for the earlier time point and maintained with gentamycin (10 μg/ml) for the duration of the experiment. At 2 and 8 h p.i., cells were washed and lysed in 1% Triton X-100. Serial dilutions were plated and colony forming units (CFUs) were counted.

### LDH sequestration assay

1.3 x 10^5^ HEK293-GFP-LC3 cells were seeded in 12-well plates (Falcon) coated with 10 μg/ml poly-D-lysine (Sigma). Two days later, the medium was replaced with 1.5 ml fresh medium and 500 μl MVs, 500 μl PBS (vehicle-control), or 500 μl medium (used for subtraction of background LDH sedimentation; Seglen et al., 2015) was added. After 3 h, Torin1 (50 nM) or DMSO (0.1%) was added, and after an additional hour BafA1 (100 nM) or DMSO (0.1%) was added. Following an additional 2 h incubation, the cells were harvested with the use of Accumax (Sigma), and the autophagic sequestration rates were determined as previously described (Seglen et al., 2015), with minor modifications. Briefly, the cells were washed twice in 10% sucrose/1% BSA followed by re-suspension in 400 μl 10% sucrose/0.2% BSA and selective electro-disruption of the plasma membrane by a single discharge of 2 kV/cm and 1.2 μF in a 1 × 1 × 5 cm electrode chamber coupled to an in-house power generator. The electro-disrupted cells were diluted with 400 μl phosphate-buffered sucrose (100 mM sodium phosphate, 2 mM dithiothreitol (DTT), 2 mM EDTA and 1.75% sucrose, pH 7.5), and whereas 200 μl was saved to measure total cellular LDH levels, 600 μl was mixed with 900 μl resuspension buffer (50 mM sodium phosphate, 1 mM EDTA, 1 mM DTT) containing 0.5% BSA and 0.01% Tween. After centrifugation at 20,000 × g for 45 min at 4°C, the pellets and the remaining 200 μl of total disruptate were exposed to one freeze-thaw cycle at −80°C, before measurement of sedimentable and total cellular LDH activity: pellets were dissolved in 300 μl resuspension buffer containing 1% Triton X-405, and the total cell disruptates were diluted three-fold with resuspension buffer containing 1.5% Triton X-405. Following a short centrifugation to eliminate cell debris (5 min at 21,000 × g), LDH activity was measured by the decline in NADH absorbance at 340 nm in a multi-analyzer (Maxmat PL-II, Erba Diagnostics, Montpellier, France) using an LDH-assay kit (Erba Diagnostics, RM LADH0126V). The percentage value of sedimentable LDH in untreated cells (t_start_) was subtracted from the percentage value obtained in experimentally treated cells (t_end_), and the resulting net value was divided by time (h) to obtain the rate of LDH sequestration (%/h) during the experimental treatment.

### Cell death

HEK293 cells were seeded in 96-well plates 1 day prior the experiment with a cell density 22,400 cells/well. Cells were pre-treated with 20 μg MVs for 2 h before adding 0.8 μg/ml purified recombinant LLO. To test for inhibition of LLO-induced cell death, 100 μM Necrostatin-1 (Nec-1, Sigma), 20 μM Z-VAD-FMK (Santa Cruz), or 1 μM SAR-405 (Apex-Bio) were used as inhibitors of necroptosis, apoptosis, or autophagy, respectively. Necroptosis was induced with 4 μM shikonin (Sigma) and apoptosis was induced with 160 ng/ml KillerTRAIL (Enzo) in combination with 20 ng/ml cycloheximide (CHX, Sigma). The cells were treated with Nec-1 and Z-VAD-FMK for 1 h prior to addition of shikonin or TRAIL in combination with CHX, respectively. Monitoring of the cells for the indicated time points (15 min and 4 h) was performed using live-cell imaging system Incucyte Zoom (Essen BioScience). In order to quantitatively determine cell viability, cells were trypsinized, stained with Trypan blue and quantified using a hemocytometer. Cell viability was presented as a percentage of viable cells.

### Analysis of propidium iodide influx

After incubating HEK293 cells (3 × 10^5^ per well) with *L. monocytogenes* MVs for 4.5 h and pure LLO (250 ng/ml) for 1 h, as described in the autophagy assay mentioned above, cells were trypsinized, collected and centrifuged at 200 × g for 5 min at 4°C. Subsequently, the cells were resuspended in 500 μl of propidium iodide (5 μg/ml final concentration) in 1xPBS supplemented with 0.5% FBS and incubated for 15 min at room temperature in the darkness. The cells were analyzed directly using LSRII FACS machine and data were analyzed with FACSDIVA software (BD Biosciences).

### Lipid extraction

Lipid extraction was conducted by modification of a previously described method (Zhao and Xu, 2010). MV samples were diluted with MeOH p.a. in a ratio of 1:10 volumes, vortexed vigorously for 1 min and immediately placed in an ultrasonicator waterbath for 2 min prior to incubation on ice for 30 min. Samples were then vortexed again (30 s) and centrifuged (13,000 × g, 15 min, 4°C) to separate extracted lipids from the precipitated proteins. 90% of the upper methanolic phase was transferrred to a new vial. Lipid extracts and protein fractions were dried in a speed vac evaporator, reconstituted in 1xPBS and loaded on SDS-PAGE for coomassie staining or analyzed by immunoblot for the presence of LLO.

### Analysis of LLO sequestration by MVs

For immunogold binding assay, EGDeΔ*hly* MVs (200 μg) were incubated with purified LLO (600 ng) for 3 h at 37°C. They were pelleted at 130,000 g for 1.5 h at 4°C. After incubating MVs with anti-LLO antibody (1:30 dilution) for 1 h at room temperature, they were washed by centrifugation and incubated with colloidal gold conjugated anti-rabbit (5 nm) antibody (1:45 dilution; SPI supplies, Sweden) for 1 h at room temperature. MVs were finally washed three times by centrifugation, resuspended in 1xPBS and analyzed by TEM. For this, grids were briefly fixed with 2.5% EM-grade Glutaraldehyde (TAAB) and negatively stained with 1.5% Uranyl acetate. Samples were examined with a JEM1230 transmission electron microscope (JEOL) operating at 80 kV. Micrographs were acquired with a Gatan Orius 830 2 k × 2 k CCD camera using Digital micrograph software. For immunofluorescence analysis, we used a previously described method with some modifications (Duperthuy et al., 2013). Briefly, MVs were incubated with LLO in the same way as for immunogold analysis. After washing step at 300,000 g for 30 min, pellet was re-suspended in primary anti-LLO antibody (1:200 in 1xPBS-Twin) and incubated for 1 h at room temperature. MVs were washed by addition of PBS-Twin and centrifugation. FITC-labeled anti-rabbit (Innovagen) was used as secondary antiserum at a final dilution of 1:3,000 for 1 h. At the end of incubation, MVs were stained using the PKH26 Red Fluorescent Cell Linker Kit (Sigma) diluted 1:500 and incubated for 5 min at room temperature prior centrifugation at 300,000 g for 30 min. MVs were then washed two more times by centrifugation before fixing on a glass slide and imaging with fluorescence microscopy. Another way to check whether MVs can bind pure toxin, avoiding centrifugation steps, is ELISA (Duperthuy et al., 2013). The wells of microtiter plates were coated with poly-D-lysine (Sigma; #P6407), washed thoroughly, and MVs (30 μl) were added on top for 1 h at 37°C. Further, they were incubated with pure LLO (1 μg/ml) for 1 h at 37°C 5% CO2. The plate was then washed with 1xPBS and to each well, 100 μl of anti-LLO antibody (1: 1,000 dilution) was added and incubated for 2 h at room temperature. Subsequently, the plate was washed three times and 100 μl of anti-rabbit IgG-HRP conjugate (1:3,000 dilution) was added to each well and the plate was incubated 1 h at room temperature. The plate was washed again three times and the enzymatic reaction was initiated by the addition of 100 μl of 3,3′, 5,5′- tetramethylbenzidine (TMB) substrate (Thermo Scientific) to each well containing peroxide. The plate was incubated at room temperature for 30 min before 100 μl of stop solution (0.16 M sulfuric acid) was added to each well. The absorbance was measured at 450 nm.

### Statistics

All data are representative of at least 3 independent experiments. *P-*values were calculated using the two-tailed unpaired Student's *t-*test and paired Student's *t*-test for the LDH sequestration data. A *p*-value of <0.05 was considered statistically significant and is denoted by “^*^,” while *p* < 0.01 is denoted by “^**^” and *p* < 0.001 is denoted by “^***^”.

## Results

### *L. monocytogenes* produces membrane vesicles (MVs) both *in vitro and in vivo*

**Figure 1:**
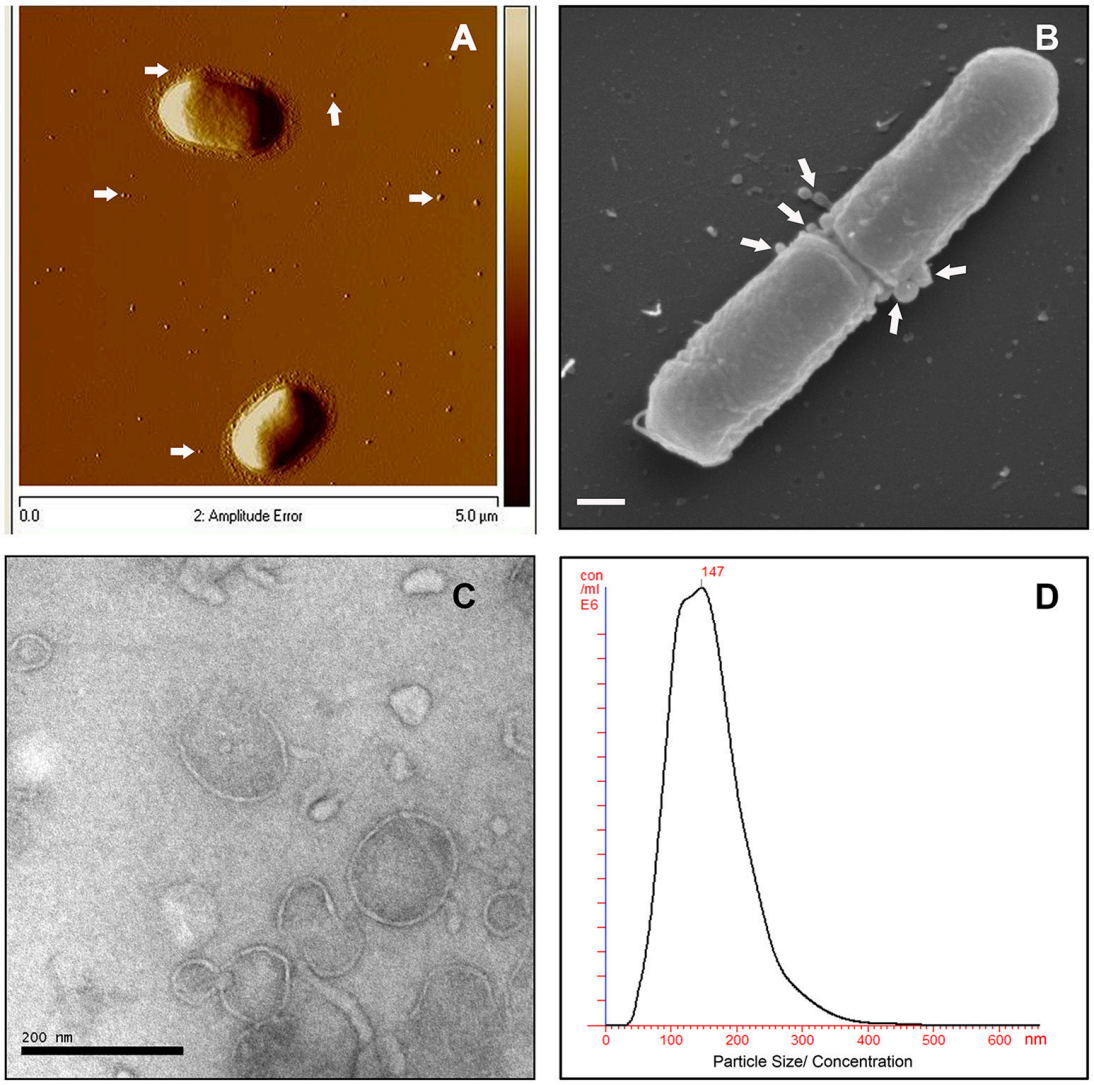
***L. monocytogenes*** **MVs production *in vitro*. (A)** Atomic force micrograph (AFM) of the EGDe strain, grown at 37°C till stationary phase. White arrows show released MVs surrounding the bacterial cells. Bar size: 5 μm. **(B)** Scanning electron micrograph (SEM) of the EGDe strain releasing MVs (indicated with white arrows) particularly at the cell division site. Bar size: 200 nm. **(C)** Transmission electron micrograph (TEM) of isolated and Optiprep density-gradient purified MVs from fraction 17. Scale bar: 200 nm. **(D)** Crude vesicle preparations were analyzed and quantified using Nanoparticle Tracking Analysis (NanoSight). Plot shows MVs size vs their concentration.

**Figure 2:**
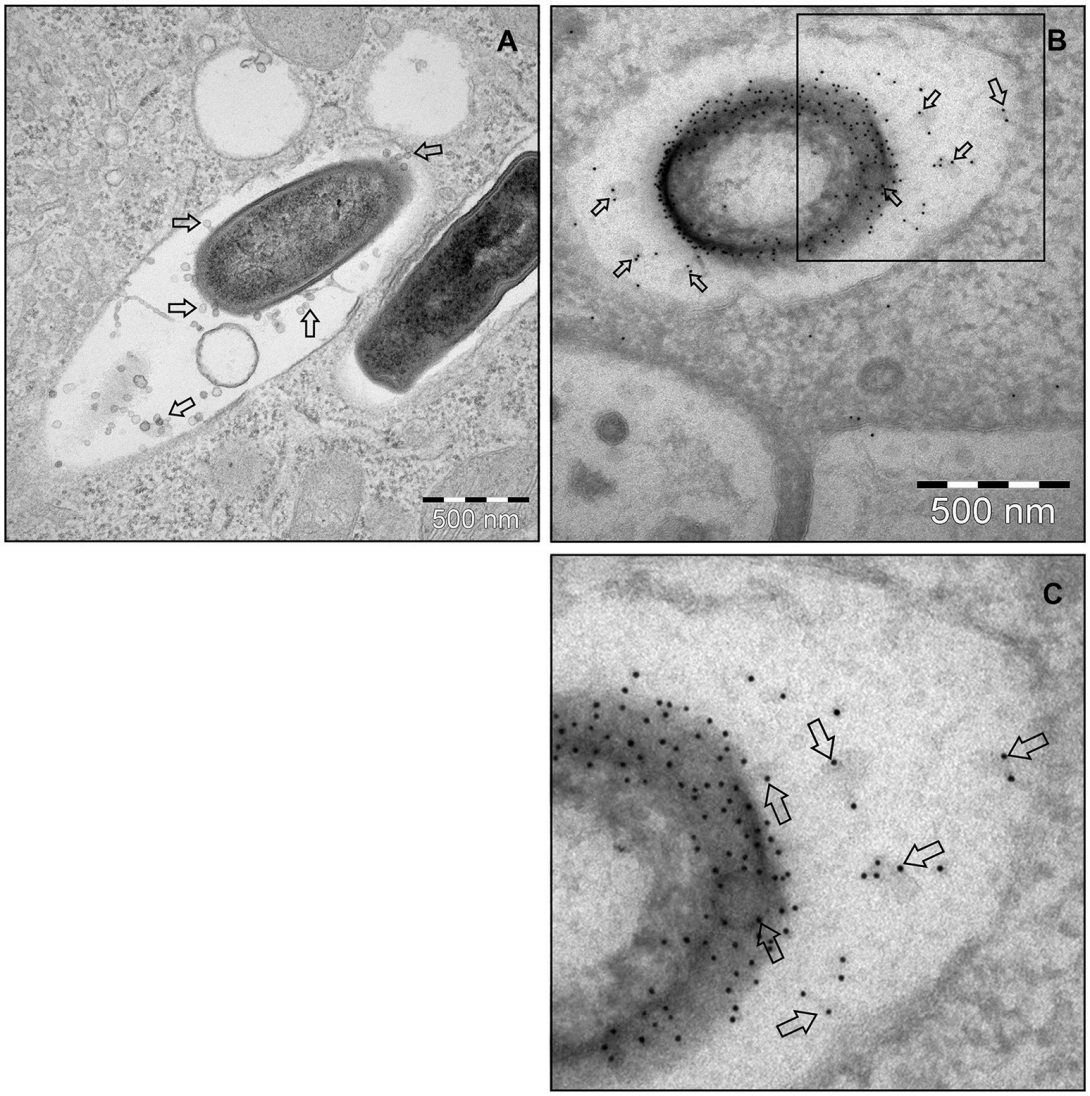
***L. monocytogenes*** **MVs production *in vivo*. (A)** Thin section electron micrograph of murine RAW264.7 macrophages infected with EGDeΔ*hly L. monocytogenes* grown to exponential phase for 90 min at the MOI 20:1. Micrograph shows phagosome containing intracellular bacterium releasing MVs (shown with arrows). Scale bar: 500 nm. **(B)** Immunogold thin section electron micrograph of murine RAW264.7 macrophages infected with the EGDeΔ*hly L. monocytogenes*. MVs released from bacterial surface inside the phagosome were detected using anti-Listeria O antiserum type I followed by protein A conjugated 10 nm gold nanoparticles (indicated with arrows). Scale bar: 500 nm. **(C)** Electron micrograph showing a magnified area in the square of Panel **(B)**.

To investigate MV production in *L. monocytogenes*, wild type strain EGDe was grown in LB media and the bacterial surface was analyzed by atomic force microscopy (AFM) or SEM. AFM and SEM analyses revealed the presence of MVs surrounding the bacterial cells with some MVs released from the bacteria (Figures 1A,B). We isolated and purified MVs released from bacterial cells grown in Luria-Bertani broth using density-gradient centrifugation and examined the obtained fractions by transmission electron microscopic analysis (TEM). Bilayered MVs were observed mainly by TEM in fractions 15–20 (Figures 1C,**4A**). Nanoparticle Tracking Analysis (Nanosight) revealed that the MVs samples were heterogeneous, having diameters ranging from 50 to 350 nm, with MVs of 147 nm being the most abundant (Figure 1D). *L. monocytogenes* is known to be a successful intracellular pathogen since it can grow and replicate inside host cells. To investigate whether *L. monocytogenes* can produce MVs inside eukaryotic cells, RAW-blue macrophages were infected with *L. monocytogenes* and subjected to cell thin-sectioning with further TEM analysis. Furthermore, immunogold labeling with anti*-Listeria* antibody was performed to confirm that vesicles released inside the phagosomes are bacteria-derived. As shown in Figure 2, MVs were observed in the vicinity of the bacterial cells inside the macrophages. Thus, *L. monocytogenes* secretes MVs both outside and inside eukaryotic cells.

### MVs are endocytosed by mammalian cells and accumulated in lysosomes

**Figure 3:**
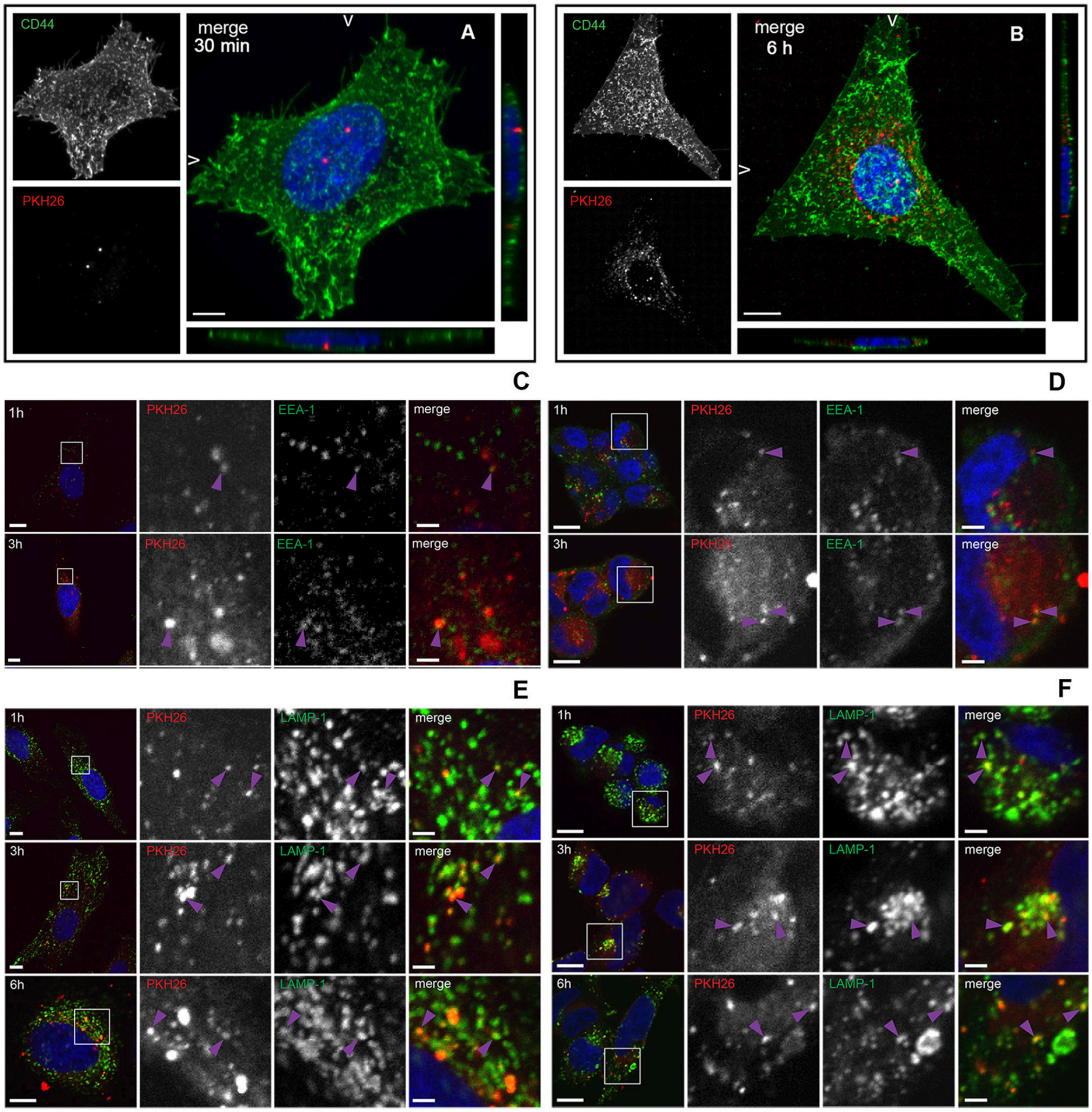
**Internalization and trafficking of MVs inside HeLa and HEK293 cells. (A,B)** Confocal microscopic images showing MVs taken up by HeLa cells after 30 min **(A)** or 6 h **(B)**. HeLa cells were treated with 100 μg of PKH26 pre-labeled MVs (red), and stained with CD44 (cell surface marker; green) and DAPI (nuclei; blue). The left panels show images from CD44 staining only (green) and PKH26 staining only (red). The right panels show merged images (maximum intensity projections and slice views). Scale bar: 10 μm. **(C–F)** Confocal microscopic images showing colocalization of MVs with early endosomes and lysosomes. HeLa cells **(C,E)** and HEK293-GFP-LC3 cells **(D,F)** were treated with 100 μg of PKH26 pre-labeled MVs (red) for different time points and stained with anti-EEA-1 antibody (green) and DAPI (blue) **(C,D)** or with anti-LAMP-1 antibody (green) and DAPI (blue) **(E,F)**. Colocalization is indicated with purple arrowheads. Left panels show an overview of the cell and the magnified fields in squares are presented in the panels to the right, where MVs and EEA-1 or LAMP-1 are shown individually and as a merged image of all three channels, as indicated. Scale bar: 10 μm (overview) and 2 μm (zoom).

To investigate whether *L. monocytogenes* MVs can be internalized by eukaryotic cells, isolated MVs were labeled with the lipid dye PKH26 and HeLa cells were incubated with labeled MVs. CD44 was used as a cell surface marker and DAPI was used for nuclei staining. The samples were collected at different time points. As shown in Figure 3A, MVs were detected inside HeLa cells already after 30 min of incubation. After 6 h of incubation, the number of internalized vesicles was greatly increased. Interestingly, MVs were concentrated in the vicinity and surrounding the nuclei of the HeLa cells after 6 h of incubation (Figure 3B). In order to investigate the fate of MVs inside the cells, we infected HeLa and HEK293-GFP-LC3 cells with MVs for 1, 3, or 6 h. Immunofluorescent microscopic analyses were performed using different intracellular markers: anti-LC3B antibody to stain phagosomes and autophagic membranes, anti-EEA1 marker to stain early endosomes and anti-LAMP-1 marker to stain lysosomes. We detected MVs colocalization with an early endosomal marker EEA-1 after 1 and 3 h treatment with MVs in both cell types (Figures 3C,D). Most of the MVs were colocalized with lysosomes after 1, 3, and 6 h in both HeLa and HEK293 cells (Figures 3E,F). However, there was no colocalization of stained MVs with phagosomes or autophagic membranes after 1, 3, or 6 h in the HeLa and HEK293 cells (Figures S1A,B). According to Pearson's correlation coefficient, colocalization of MVs and lysosomes increased in a time-dependent manner for HeLa, but not for HEK293 cells (Figure S1C). That might be due to a different MVs uptake ability of the different cell types, which suggests that HeLa cells have slower uptake of MVs than HEK293 cells. Together, these data indicate that *L. monocytogenes* MVs are internalized into mammalian cells and further accumulated in the lysosomes.

### Listeriolysin O is mainly integrated within the MVs

**Figure 4:**
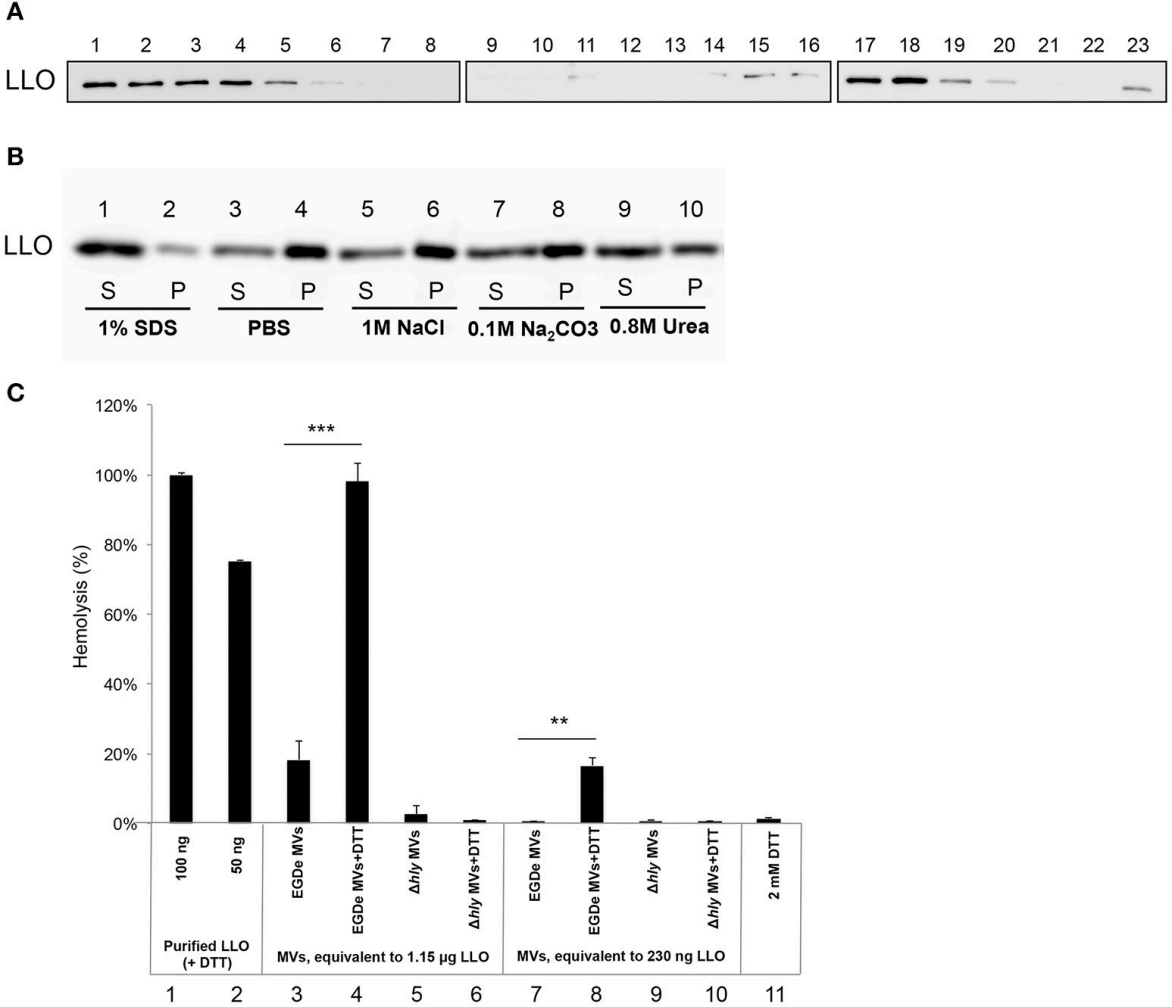
**Listeriolysin O localization and activity inside the MVs**. **(A)** Immunoblot detection of listeriolysin O (LLO) in density gradient fractions of isolated and purified MVs. Fractions are numbered from left to right (1–23) according to increasing density. Fractions 1-5 containing soluble LLO do not show MVs under TEM; fractions 15-20 contain MVs as shown in Figure 1C. Polyclonal anti-LLO antiserum was used to detect LLO at 56 kDa using SDS-PAGE and immunoblot analysis of all the fractions. **(B)** LLO is tightly associated with MV structure. Dissociation assay was performed using 1 M NaCl, 0.1 M Na_2_CO_3_, 0.8 M urea or 1% SDS. After incubation, MVs were ultracentrifuged, and supernatant (S) and pellet (P) fractions were analyzed by immunoblot using a polyclonal anti-LLO antiserum. **(C)** MV-associated LLO shows no hemolytic activity unless exposed to reducing conditions. Hemolytic activity of MVs from EGDe (wt) and the Δ*hly* strain in comparison with purified LLO containing DTT. Goat erythrocytes (10%) were incubated either with LLO (100 ng or 50 ng) or with MVs (containing 1.15 μg LLO or 230 ng LLO) for 3 h at 37°C. Where indicated, MVs were pre-incubated with a reducing agent, 2 mM DTT, for 20 min at 25°C. Hemolytic activity of 100 ng LLO was normalized up to 100%; 2 mM DTT was used as a negative control. The mean with SD is shown. Results represent three independent experiments. ^**^P < 0.01, ^***^P < 0.001, Student's *t-* test.

LLO was previously found to be associated with *L. monocytogenes* MVs (Lee et al., 2013). To examine whether LLO was secreted from bacteria in a tight association with MVs, MVs were purified using Optiprep density-gradient centrifugation, and fractions were analyzed. Immunoblot analysis revealed that a large amount of LLO was present in fractions 15–20, the fractions where MVs were observed by TEM (Figures 1C,4A). LLO was also detected in fractions 1–5, which did not contain MVs (Figure 4A). These results suggest that a significant proportion of LLO is secreted in tight association with MVs released from *L. monocytogenes*. To further investigate whether LLO was located inside the MVs or associated on the surface of the MVs, we performed a dissociation assay. As shown in Figure 4B, the majority of the LLO protein was recovered in the MV pellet after treatment with PBS, 1M NaCl, or 0.1M Na_2_CO_3_ (Figure 4B, lanes 3–8), while treatment with 0.8M urea partly dissociated LLO from the MVs resulting in a lower LLO content in the pellet (Figure 4B, lanes 9–10). Only upon SDS solubilization of the MVs, the LLO protein was released and mostly remained in the supernatant after ultracentrifugation (Figure 4B, lanes 1 and 2). These results suggest that the majority of MV-associated LLO is either enclosed by the MV structure or embedded in the vesicle membrane, while a portion of LLO secreted as a free toxin appears to be subsequently re-associated on the surface of MVs.

### Membrane vesicles show hemolytic activity under reducing conditions

LLO is a known pore-forming CDC toxin, which causes hemolysis of erythrocytes by membrane perforation. Therefore, we next tested the hemolytic activity of LLO-containing MVs. The amount of MV-associated LLO was estimated by immunoblot analysis of MV extracts using anti-LLO antiserum and comparing the intensity of the immuno-reactive bands with those of known concentrations of purified recombinant LLO. Using the standard curve we found that 10 μl of MVs (equal to 5 μg of MVs) contained ~230 ng of LLO (i.e., 23 μg/ml; Figure S2). MVs containing 230 ng of LLO did not cause hemolysis, and only partial hemolysis (18%) was observed when MVs containing 1.15 μg of LLO were used (Figure 4C, lane 3 and 7). Previous studies showed that LLO is only hemolytic in the reduced form (Westbrook and Bhunia, 2000). Therefore, we were interested in examining whether MV-associated LLO treated with a reducing agent (DTT) would affect hemolysis. After DTT treatment of MVs, partial hemolytic activity was observed already with 230 ng of MV-associated LLO (Figure 4C, lane 8). MVs containing a higher amount of LLO (1.15 μg) showed full hemolytic activity, equivalent to that obtained with 100 ng of purified LLO (Figure 4C, lane 4 vs. lanes 1 and 2). As expected, MVs from the Δ*hly* strain (which lacks the gene encoding LLO) did not show any hemolytic activity (Figure 4C, lanes 5–6 and 9–10). Taken together, these results suggest that LLO resides in the MVs in a predominantly oxidized, inactive form and that LLO associated with the outer surface of MVs can be activated under reducing conditions, causing dose-dependent hemolysis of erythrocytes.

### *L. monocytogenes* MVs protect against pore-forming toxin-induced autophagy

**Figure 5:**
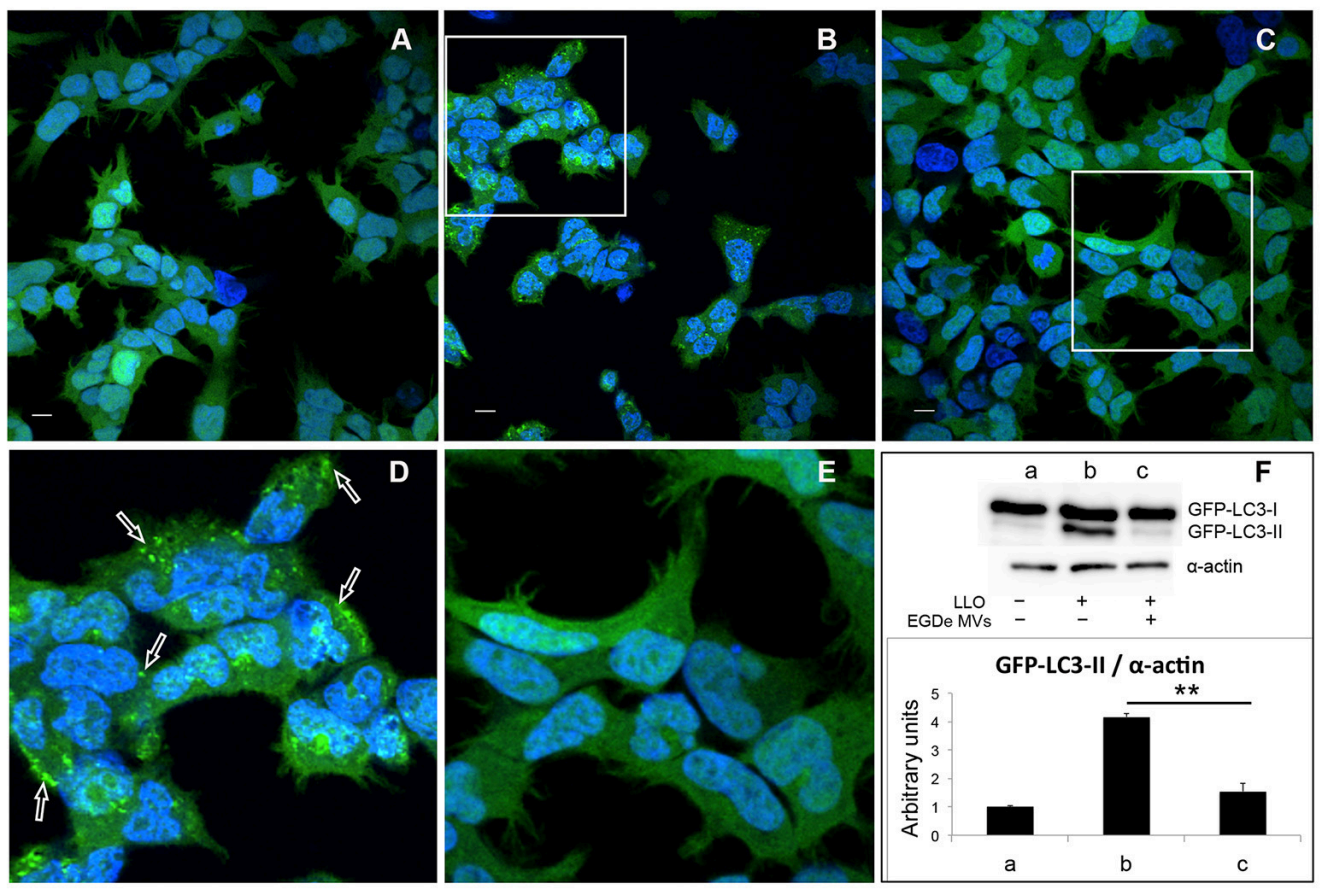
**MVs inhibit autophagy induced by purified LLO**. HEK293-GFP-LC3 cells were incubated with 200 μg MVs (isolated from EGDe bacterial cultures grown for 40 h) for 4.5 h before autophagy was induced with 250 ng of purified LLO for 1.5 h. Confocal microscopic images show **(A)** mock-treated cells (PBS), **(B)** cells treated with only LLO and **(C)** cells treated with MVs + LLO. **(D)** is a magnified area of the white square in **(B)**, and **(E)** is magnified area of the white square in **(C)**. Scale bar: 10 μm. Green dots represent GFP-LC3 puncta (arrows). **(F)** HEK293-GFP-LC3 cells were incubated with MVs and LLO in the same way as for confocal microscopy. Host cell lysates were analyzed for GFP-LC3 lipidation by immunoblot using monoclonal GFP antiserum (upper panel). The membrane was reprobed using anti-α-actin antibody as an internal control. The ratio of GFP-LC3-II to α-actin is presented in arbitrary units (lower panel). Immunoblots show **(a)** mock-treated cells, **(b)** cells treated with only LLO, and **(c)** cells treated with MVs + LLO. Data was quantified, normalized and shown from three independent experiments. ^**^P < 0.01, Student's *t-*test.

Earlier studies demonstrated that a variety of bacterial pore-forming toxins (PFTs), such as streptolysin O (SLO), perfringolysin, *V. cholerae* cytolysin (VCC) and LLO, can induce autophagy (Nakagawa et al., 2004; Gutierrez et al., 2007; Meyer-Morse et al., 2010). Given that MV-associated LLO is relatively hemolytically inactive, we further aimed to test whether LLO-containing MVs were able to induce autophagy. The autophagy assay was performed using HEK293 cells stably expressing the autophagic marker GFP-LC3. Upon induction of autophagy, cytosolic LC3 (LC3-I) is conjugated to phosphatidylethanolamine in autophagy-related membranes forming the lipidated form of LC3 (LC3-II). Concentration of GFP-LC3-II on pre-autophagosomal and autophagosomal membranes can be visualized as bright fluorescent GFP-LC3 puncta within cells. Moreover, the lipidated and unlipidated forms of LC3 can be distinguished by SDS-PAGE mobility shift, where LC3-II has a faster mobility than LC3-I (Klionsky et al., 2016). In contrast to treatment with recombinant LLO, which strongly induced the formation of GFP-LC3 puncta (Figures 5B,D), LLO-containing MVs had no effect (Figures S3A,B). This indicated that MV-associated LLO is unable to induce autophagy either because the associated LLO is largely inactive in terms of pore-forming ability and/or because MVs protect against the autophagy-inducing effects of LLO. With respect to the latter, we strikingly found that MV pre-treatment resulted in a near complete abrogation of LLO-induced GFP-LC3 puncta formation (Figures 5C,E). Furthermore, we found that LLO-induced conversion of GFP-LC3-I to GFP-LC3-II was strongly reduced by pre-treatment with MVs (Figure 5F). Not only MVs isolated from the wild-type strain inhibited autophagy but also MVs from the EGDeΔ*hly* strain (Figure S3D), indicating that MV-associated LLO itself was not involved in this inhibitory effect. EGDeΔ*hly* MVs alone did not induce autophagy (Figure S3C). Thus, MVs appear to very efficiently inhibit LLO-induced autophagy. We also observed that MVs isolated from bacterial cultures in LB broth after 16 h of growth had similar potent autophagy-inhibiting activity as MVs isolated after 40 h of growth [compare Figure 5 (MVs isolated after 40 h) with Figure S4 (MVs isolated after 16 h of growth)], demonstrating that autophagy inhibition by MVs was not dependent on the bacterial growth phase. In order to assess whether MVs possibly would affect the stability of LLO, we incubated purified LLO with MVs or only PBS *in vitro* for 1.5 h, and examined LLO stability by immunoblot using anti-LLO antibody (Figure S5A). There was no apparent degradation of LLO caused by co-incubation with MVs indicating that MVs do not reduce autophagy by degrading LLO.

**Figure 6:**
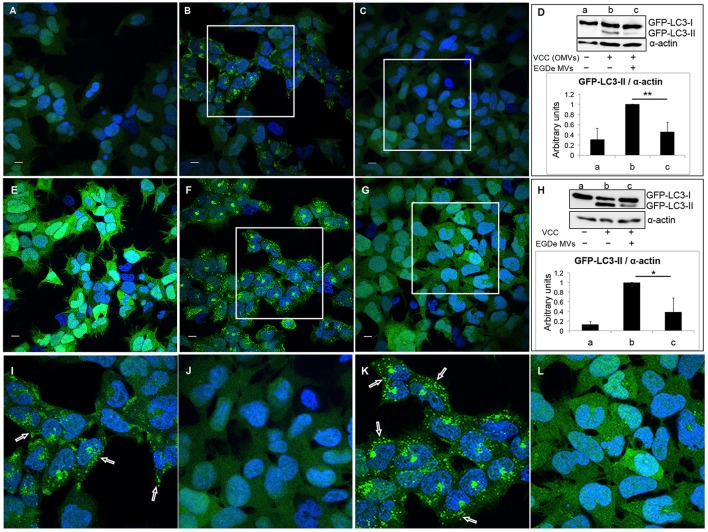
***Listeria monocytogenes*** **MVs inhibit autophagy induced by pore-forming toxin (VCC) associated with outer membrane vesicles (OMVs) of *V. cholerae***. HEK293-GFP-LC3 cells were incubated simultaneously with 250 μg MVs from the *L. monocytogenes* EGDe strain and 200 μg of *V. cholerae* V:5/04 OMVs (autophagy inducer) for 6 h **(A–D)** or 2.4 nM of purified *V. cholerae* cytolysin (VCC) for 5 h **(E–H)**. Confocal images show **(A,E)** mock-treated (PBS) control cells, **(B)** cells treated with only *V. cholerae* V:5/04 OMVs and **(C)** cells treated with *V. cholerae* V:5/04 OMVs and EGDe MVs, **(F)** cells treated with only VCC and **(G)** cells treated with VCC and EGDe MVs. **(I)** and **(J)** represent magnified areas of the white squares in **(B)** and **(C)**, respectively; **(K)** and **(L)** represent magnified areas of the white squares in **(F)** and **(G)**, respectively. Scale bar: 10 μm. Green dots represent GFP-LC3 puncta, indicated with white arrows. The results represent at least three independent experiments. **(D,H)** Host cell lysates were analyzed for conversion of GFP-LC3-I (cytosolic) to GFP-LC3-II (membrane-conjugated) form by immunoblot using monoclonal GFP antiserum (upper panels). The membrane was reprobed using anti-α-actin antibody as an internal control. **(D)** Immunoblots show **(a)** mock-treated cells, **(b)** cells treated with only *V. cholerae* OMVs, and **(c)** cells treated with MVs from both bacteria. **(H)** Immunoblots show **(a)** mock-treated cells, **(b)** cells treated with only VCC, and **(c)** cells treated with VCC and EGDe MVs. The ratio of GFP-LC3-II to α-actin was quantified from three independent experiments, normalized and presented in arbitrary units (lower panels). ^**^P < 0.01, ^*^P < 0.05, Student's *t-*test.

Next, we tested whether the autophagy-inhibitory effect of MVs is specific to LLO-induced autophagy or if the same effect can be observed with other PFTs. For this we used another β–class PFT, *Vibrio* cytolysin (VCC), which was previously described as an autophagy inducer (Gutierrez et al., 2007; Elluri et al., 2014). Outer membrane vesicles (OMVs) containing VCC from *V. cholerae* strain V:5/04 were incubated with HEK293-GFP-LC3 cells for 6 h in the absence (Figures 6A,E) or presence (Figures 6C,G,J,L) of MVs from the *L. monocytogenes* EGDe strain. As expected, VCC-containing *V. cholerae* OMVs induced a large number of GFP-LC3 puncta (Figures 6B,I) as well as LC3 lipidation (Figure 6D, lanes a and b). Strikingly, *L. monocytogenes* MVs strongly reduced both GFP-LC3 puncta formation and LC3 lipidation induced by *V. cholerae* OMVs (Figures 6C,D lanes c and j). To exclude the possibility that internalization of both types of MVs can alter the ability of *V. cholerae* MVs to induce autophagy, we used purified VCC toxin to induce autophagy (Figures 6F,K). Autophagy induced by VCC-containing *V. cholerae* OMVs or by purified VCC was equally well abrogated by *L. monocytogenes* MVs as observed by confocal microscopy and LC3-immunoblot analyses (Figures 6G,H,L). To test whether *L. monocytogenes* MVs can directly interact with and degrade OMV-associated VCC, membrane vesicles from both bacterial species were mixed together *in vitro* and the mixture was incubated at 37°C for 6 h. OMV-associated VCC did not exhibit any degradation in the presence of *L. monocytogenes* MVs, as assessed by immunoblot analysis (Figure S5B). Taken together, our data indicate that although *L. monocytogenes* MVs contain abundant LLO, the LLO-containing MVs are unable to induce autophagy. Instead, *L. monocytogenes* MVs show a remarkable potent protective effect against autophagy induced by pore-forming toxins.

### *Listeria* MVs suppress Torin1-induced autophagy

**Figure 7:**
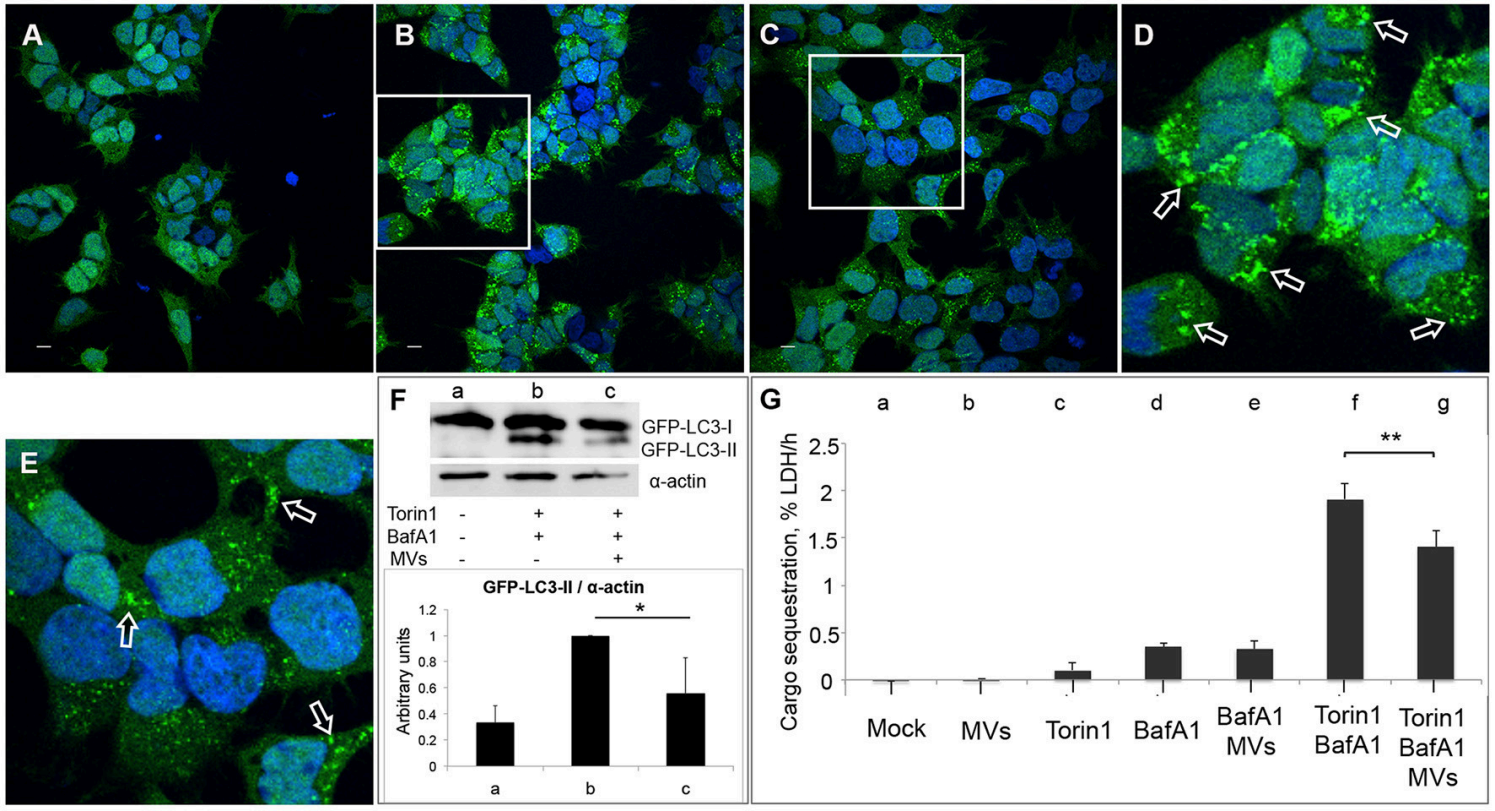
**MVs inhibit Torin1-stimulated autophagy**. HEK293-GFP-LC3 cells were incubated with 250 μg MVs for 3 h prior to adding Torin1 (50 nM) in the presence of Bafilomycin A1 (BafA1; 100 nM) for 3 h to stimulate autophagosome formation. Confocal images show **(A)** mock-treated (PBS) control cells, **(B)** cells treated with Torin1 and BafA1, **(C)** cells treated with MVs followed by Torin1 + BafA1 treatment. **(D,E)** represent magnified areas of the white squares in **(B)** and **(C)**, respectively. Scale bar: 10 μm. Green dots represent GFP-LC3 puncta, indicated with arrows (only a few of the puncta are indicated with arrows for the purpose of showing examples. Many of the dots are so large that they are partly merging with one another—a common feature observed after treatment with an autophagy inducer together with BafA1). **(F)** Immunoblot shows LC3 lipidation profile of: **(a)** mock-treated cells, **(b)** cells treated with Torin1 + BafA1, and **(c)** cells treated with MVs + Torin1 + BafA1 (upper panel). The membrane was reprobed using anti-α-actin antibody as an internal control. The ratio of GFP-LC3-II to α-actin was quantified from three independent experiments, normalized and presented in arbitrary units (lower panel). **(G)** LDH sequestration assay of HEK293-GFP-LC3 cells incubated with **(a)** Mock (PBS and DMSO), **(b)** MVs, **(c)** Torin1, **(d)** BafA1, **(e)** MVs + BafA1, **(f)** Torin1 + BafA1, and **(g)** MVs + Torin1 + BafA1. Values represent mean autophagic sequestration rates (% LDH/h) ± SEM from three independent experiments. ^**^P < 0.01, ^*^P < 0.05, Student's *t-*test.

Previous studies showed that in the presence of low levels of LLO, *L. monocytogenes* is targeted by LC3-associated phagocytosis (LAP) inside host macrophages, which helps bacteria to establish a survival niche within spacious *Listeria*-containing phagosomes (SLAPs; Lam et al., 2013). The non-canonical LAP pathway is characterized by direct conjugation of LC3 to the single phagosomal membrane (Shibutani and Yoshimori, 2014). Therefore, we sought to determine whether the autophagy-inhibitory effect of *L. monocytogenes* MVs could be restricted to LAP or whether MVs more broadly can inhibit canonical autophagy. For this purpose, we stimulated autophagosome formation in HEK293-GFP-LC3 cells using the mTOR-inhibitor Torin1 in combination with Bafilomycin A1 (BafA1). Torin1 induces classical canonical autophagy, while BafA1 prevents the degradation of autophagosomes by blocking the V-ATPase proton pump, leading to de-acidification of the lysosomes and thus the inactivation of acid lysosomal hydrolases (Yoshimori et al., 1991). Additionally, BafA1 can inhibit the fusion between autophagosomes and lysosomes. Since BafA1 inhibits the degradation of LC3-II as well as the contents of autophagosomes, the process of autophagosome formation can be studied by monitoring accumulation of LC3 puncta, LC3-II levels and autophagic cargo in the presence of BafA1 (Klionsky et al., 2016). We pre-treated HEK293-GFP-LC3 cells with *L. monocytogenes* MVs for 3 h prior to an additional 3 h treatment in the presence of Torin1 and BafA1. Confocal microscopy revealed that MVs substantially decreased Torin1/BafA1-induced accumulation of GFP-LC3 puncta (Figures 7A–E). Furthermore, MVs also markedly decreased Torin1/BafA1-induced accumulation of GFP-LC3-II (Figure 7F). Despite the advantages of the LC3 assay for monitoring autophagy, the method has some limitations. For example, the LC3 protein may aggregate in an autophagy-independent manner, and although canonical macroautophagy is often associated with autophagosomal-lysosomal LC3 flux, it does not require LC3 (Szalai et al., 2015; Klionsky et al., 2016). Therefore, as an alternative, we used a recently well-validated quantitative method to monitor macroautophagic cargo sequestration (Seglen et al., 2015; Szalai et al., 2015; Klionsky et al., 2016). This method measures the transfer of the *bona fide* macroautophagic cargo marker, lactate dehydrogenase (LDH) enzyme, from the cytosolic to the organelle-containing cell fraction (Seglen et al., 2015). We found that *L. monocytogenes* MVs did not influence the level of basal autophagy in HEK293 cells treated with BafA1 alone. However, MVs significantly inhibited Torin1/BafA1-induced macroautophagic LDH sequestration (Figure 7G). Taken together, our results from confocal microscopy, immunoblotting and the autophagic cargo sequestration assay indicate that *L. monocytogenes* MVs suppress the level of canonical autophagy stimulated by the mTOR-inhibitor Torin1.

### Autophagy suppression by MVs is associated with reversal of LLO-induced inhibition of mTORC1 activity

**Figure 8:**
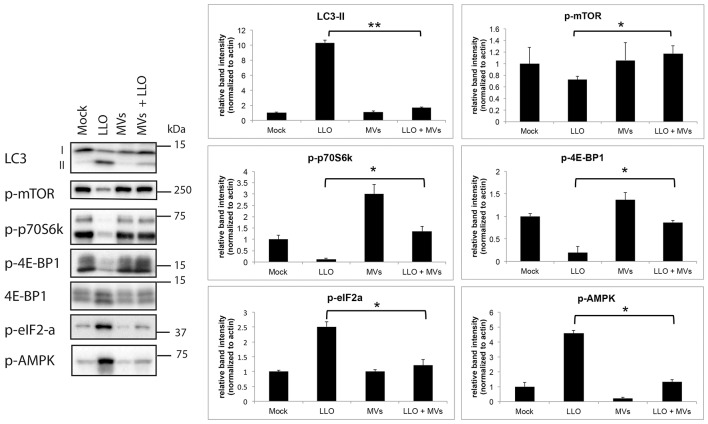
***L. monocytogenes*** **MVs reverse LLO-induced inhibition of mTORC1 activity in HEK293 cells**. Western blot of HEK293-GFP-LC3 cells treated with (1) mock, (2) purified LLO, (3) MVs, and (4) MVs and LLO, for 6 h in total. Cell protein extracts were blotted and analyzed for levels of endogenous LC3-I and LC3-II, phosphorylated mTOR (p-mTOR; Ser2448), phosphorylated p70 ribosomal S6 protein (p-p70S6K; Thr389), phosphorylated (Thr37/46)- and total 4E-BP1, phosphorylated AMPK (p-AMPK; Thr172) and phosphorylated eIF2-α (p-eIF2-α; Ser51); left panel. LC3-I and -II denote the nonlipidated and lipidated forms, respectively. The levels of phosphorylated proteins were normalized to actin; right panel. Results are quantified from three independent experiments and presented in arbitrary units. Statistically significant difference is shown between cells treated with purified LLO and cells treated with MVs + LLO. ^**^P < 0.01, ^*^P < 0.05, Student's *t-*test.

Earlier studies have demonstrated that several microbes can manipulate the autophagy pathway at the molecular level as a strategy to establish persistent infection and/or colonization (Campoy and Colombo, 2009). *L. monocytogenes* also utilizes multiple mechanisms to avoid targeting by autophagy during colonization of the host (Birmingham et al., 2008b; Dortet et al., 2011; Tattoli et al., 2013). To gain more insight into how MVs from *L. monocytogenes* inhibit autophagy induced by purified LLO, we examined the activity of the master negative regulator of autophagy, the mTOR complex 1 (mTORC1; Laplante and Sabatini, 2009). To this end, we determined the phosphorylation levels of mTORC1 and two direct targets of mTORC1, 4E-BP1 and p70S6K, by immunoblot analysis. As shown in Figure 8, levels of phosphorylated mTOR, 4E-BP1, and p70S6K were strongly reduced by treatment with purified LLO in HEK293 cells. Moreover, and strikingly, pretreatment with *L. monocytogenes* MVs strongly abrogated this inhibitory effect of LLO. In the same experiment, MVs also markedly reversed LLO-induced lipidation of endogenous LC3 (Figure 8), confirming our previous indications of the anti-autophagic function of MVs. Phosphorylation of eIF2α was shown earlier to be important for accumulation of autophagosomes and autophagy induction (Kloft et al., 2010). In line with this report, we found that LLO strongly stimulated phosphorylation of AMPK and eIF2α, and intriguingly, this was completely blocked by co-treatment with MVs (Figure 8). Together, these results strongly suggest that the mechanism whereby MVs block LLO-induced autophagy is related to reversal of LLO-mediated reduction of mTORC1 activity, presumably via reversal of AMPK activation, and to inhibition of LLO-induced phosphorylation of eIF2α.

### MV-associated protein(s) inhibit LLO-induced autophagy

**Figure 9:**
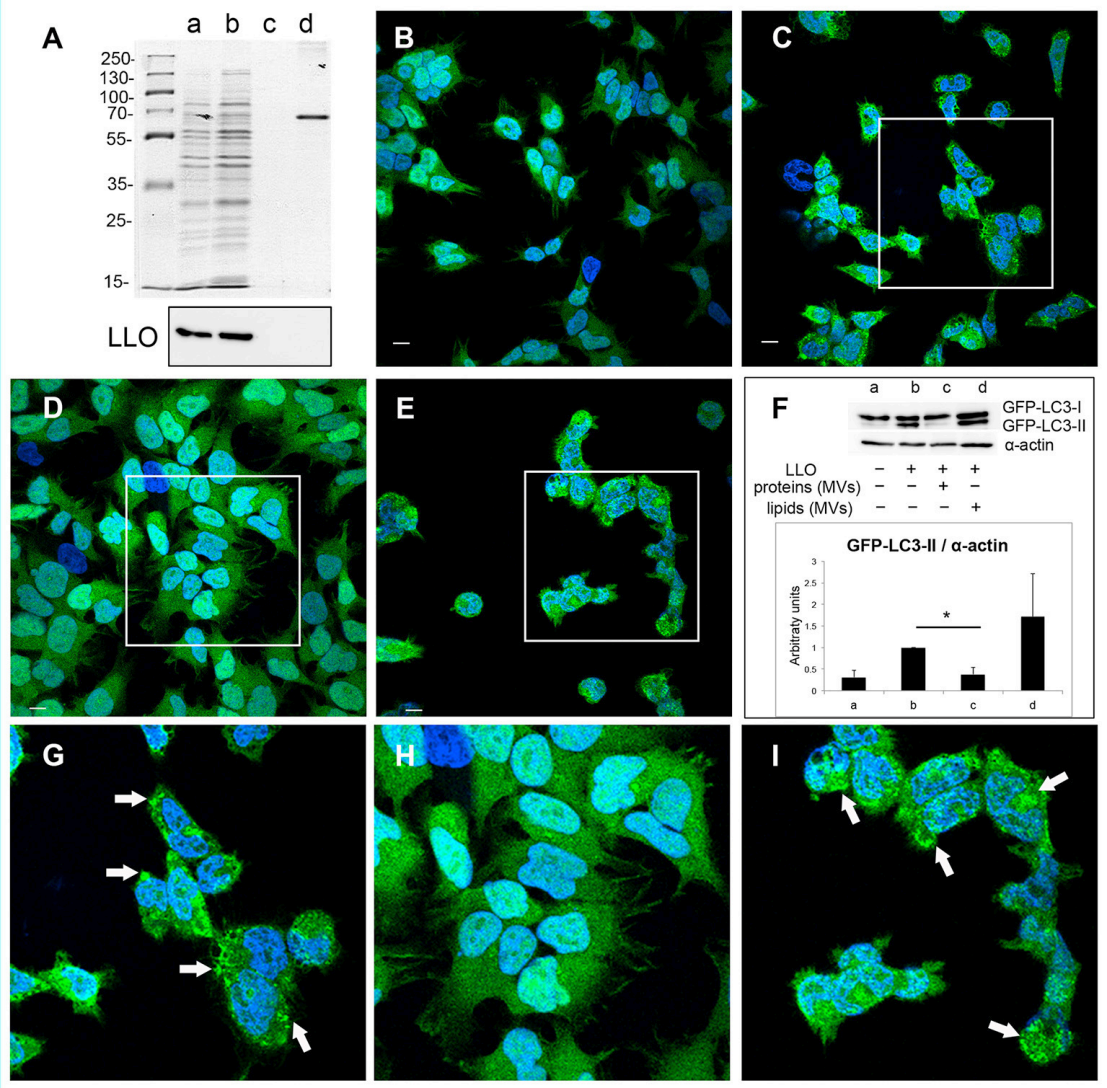
***L. monocytogenes*** **MV-associated proteins inhibit LLO-induced autophagy. (A)** Extracted proteins and lipids from *L. monocytogenes* MVs are shown on PageBlue stained polyacrylamide SDS gel: a, crude MV sample; b, extracted proteins; c, extracted lipids; d, 2.5 μg BSA control (upper panel). Protein contamination of lipid extracts was checked by immunoblotting using an anti-LLO antibody (lower panel). Confocal microscopy analysis represent: **(B)** mock-treated (PBS) control cells, **(C)** LLO-treated cells, **(D)** cells pre-treated with protein extracts from *L. monocytogenes* MVs prior LLO addition, **(E)** cells pre-treated with lipid extracts from *L. monocytogenes* MVs prior LLO addition, **(G)** represents magnified area of the white square in **(C)**, **(H)** represents magnified area of the white square in **(D)**, **(I)** magnified area of the white square in **(E)**. Scale bar: 10 μm. Green dots represent GFP-LC3 puncta indicated with white arrows. The ratio of GFP-LC3-II to α-actin **(F)** was quantified from three independent experiments, normalized and presented in arbitrary units. ^*^P < 0.05, Student's *t-*test.

In order to elucidate which components of MVs, proteins or lipids or both, are necessary for the observed inhibition of pore-forming toxin activity, we performed extraction of lipids and proteins from the MV samples. Lipids extracts were examined by PageBlue staining and immunoblotting, using anti-LLO antiserum to confirm absence of protein contamination (Figure 9A). As a positive control, we used BSA (2.5 μg). The extracted fractions of proteins and lipids were tested for their ability to inhibit LLO-induced autophagy. Autophagy was stimulated by pure LLO as described earlier (Figures 5, 8), and as shown again in these experiments by appearance of GFP-LC3 puncta (Figures 9B,C,G) and elevation of LC3-II levels (Figure 9F). As monitored by confocal microscopy of GFP-LC3 puncta, lipid extracts did not exhibit autophagy reduction (Figures 9E,I), while protein extracts totally abrogated GFP-LC3 puncta formation (Figures 9D,H). This was further confirmed by the data demonstrating reduction in GFP-LC3-I conversion to GFP-LC3-II (Figure 9F). Thus, our findings indicate that only MV-associated proteins, and not lipids, were essential for the inhibition of pore-forming toxin-induced autophagy. We are currently investigating which proteins carried by MVs are directly involved in this inhibition.

### MVs contribute to the intracellular survival of *L. monocytogenes*

**Figure 10:**
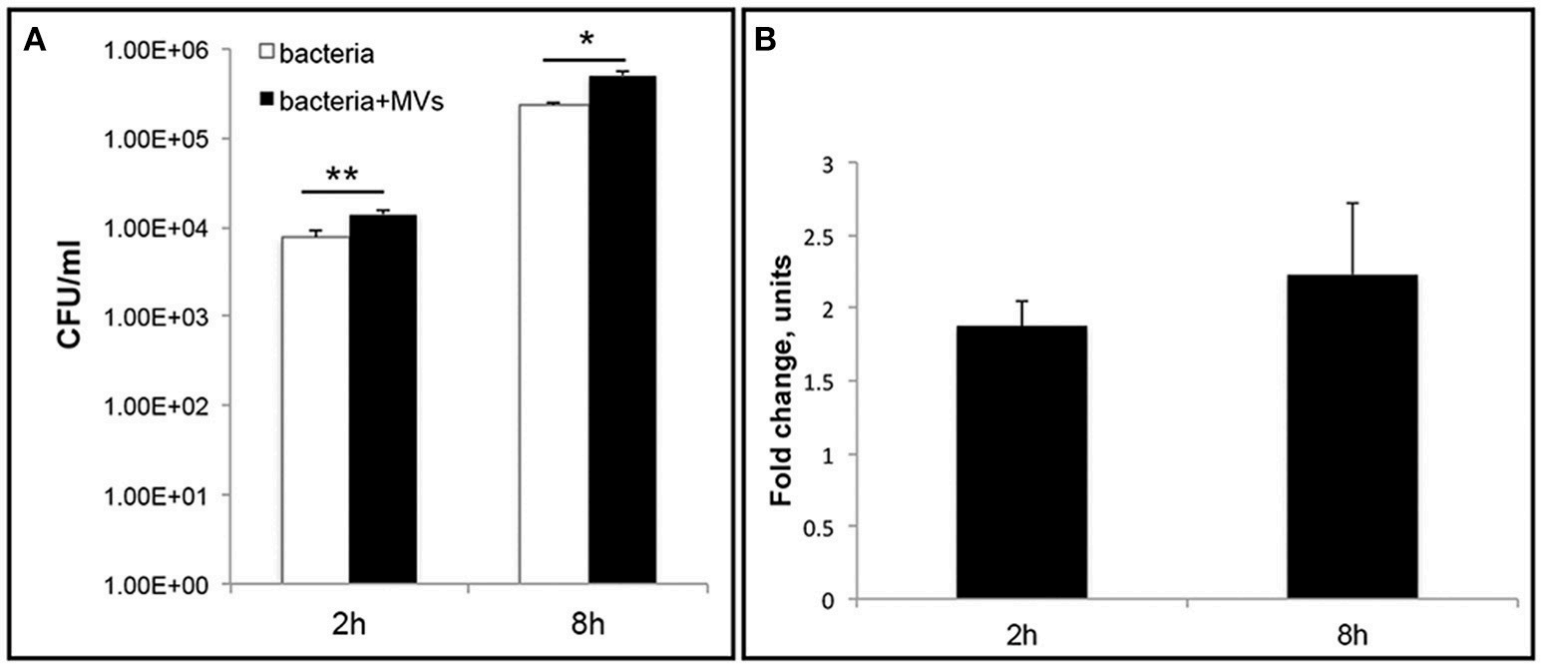
**Vesicles promote *L. monocytogenes* intracellular survival**. Mouse embryonic fibroblasts (MEFs), were pre-incubated with MVs or 1xPBS for 5 h before infection with wild type *L. monocytogenes* at an MOI of 10. After 2 h or 8 h p.i., cells were lysed and intracellular bacteria were plated to determine CFUs **(A)** The data are presented on a log scale and represent four independent experiments, error bars show ± *SD*. Asterisks indicate a significant difference, ^*^P < 0.05, ^**^P < 0.01, according to Student's *t-* test. **(B)** Survival ratio of intracellular bacteria with MVs addition to intracellular bacteria without MVs addition, represented as fold-change after 2 and 8 h of infection.

To further investigate the role of MVs during *L. monocytogenes* infection, we performed an intracellular bacterial survival assay with MEFs. MVs were incubated with MEFs for 5 h to allow MVs to be taken up by the cells before bacterial infection for 2 or 8 h. Interestingly, we observed a significant ~two-fold increase in the number of bacteria inside the MEFs pre-incubated with MVs compared to the number of intracellular bacteria without MVs pre-treatment (Figure 10). This phenomenon was observed after 2 h and after 8 h of infection. Our results suggest that MVs may promote *L. monocytogenes* survival inside eukaryotic cells. Alternative explanation can be an increased phagocytosis or an intracellular bacterial replication. To investigate further biological significance and role of MVs during *L. monocytogenes* infection, it will be relevant to use additional infection models (e.g., chicken embryos or mice).

### MVs protect host cells from LLO-induced lytic cell death

**Figure 11:**
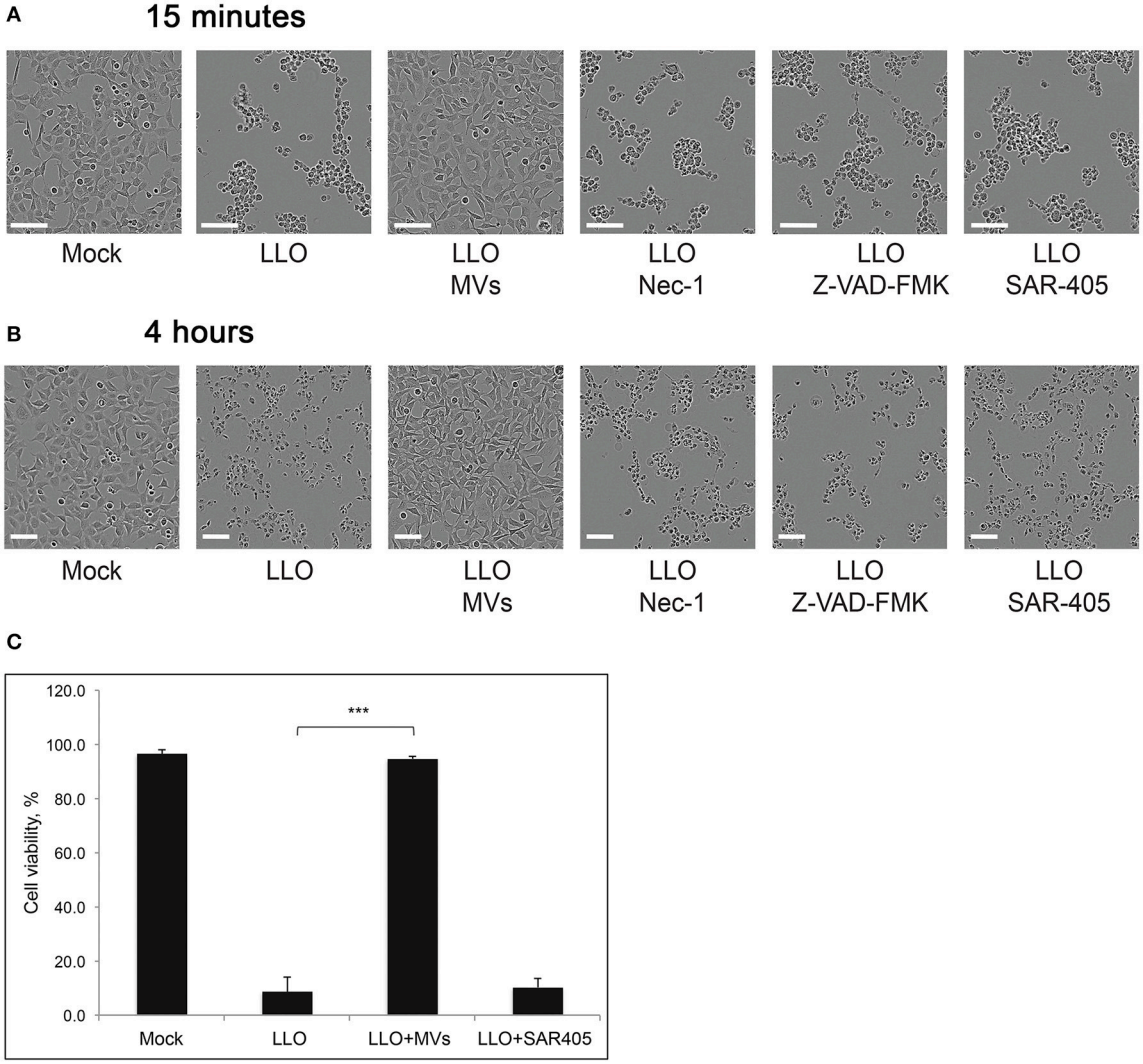
***L. monocytogenes*** **MVs protect HEK293 cells from LLO-induced necrosis**. HEK293 cells were incubated with purified LLO for **(A)** 15 min or **(B)** 4 h at a high concentration to induce cell death. Protection from cell death was tested by adding MVs (20 μg) 2 h prior to LLO treatment, and Necrostatin-1 (Nec-1, 100 μM), Z-VAD-FMK (20 μM), or SAR-405 (1 μM) 1 h prior to LLO treatment. Cells were monitored by live-cell imaging for the indicated time-points. Scale bar: 100 μm. **(C)** For a quantitative analysis of MV protective effect on HEK293 cell viability, cells were mock pre-treated, or pre-treated with *L. monocytogenes* MVs (20 μg) for 2 h or the autophagy inhibitor SAR-405 (1 μM) for 1 h in a 96-well plate. Cell death was induced with purified LLO for 4 h. Cell survival was determined using the Trypan blue viability assay, normalized to mock treated cells as 100% of survival. Results represent three independent experiments. ^***^P < 0.001, Student's *t-*test.

Increased bacterial survival and replication in the presence of MVs could be explained if MVs have a protective effect on the host cell. Previously, it was discovered that LLO could induce cell death in primary immune cells and several other cell types (Carrero et al., 2004, 2008; Aroian and van der Goot, 2007; González-Juarbe et al., 2015). In agreement, we observed that LLO in a high concentration (0.8 μg/ml) elicited massive cell death in HEK293 within 4 h of treatment (Figures 11B,C). The effect of LLO occurred very rapidly, as indicated by drastic cell rounding already after 15 min (Figure 11A). Strikingly, MVs completely protected cells from the deleterious effects of LLO at both early and late time-points (Figure 11). In order to determine which type of cell death is initiated by purified LLO and thus which type of cell death is blocked by MVs, we used the necroptosis inhibitor Necrostatin-1 (Han et al., 2007), the pan-caspase inhibitor Z-VAD-FMK (Ahmad and Shi, 2000) and the autophagy inhibitor SAR-405 (Pasquier, 2015). As shown in Figures 11A,B, these inhibitors had no impact on cell morphology after 15 min or 4 h of LLO treatment. This would be compatible with LLO inducing direct HEK293 cell lysis, rather than eliciting necroptotic, apoptotic, or autophagic cell death. In order to verify the efficiency of the inhibitors, we specifically induced necroptosis with shikonin (Han et al., 2007) and apoptosis with cycloheximide (CHX; an inhibitor of protein translation) in combination with TRAIL (Ahmad and Shi, 2000). Indeed, necrostatin-1 and Z-VAD-FMK strongly abolished the effects of shikonin and TRAIL+CHX, respectively (Figure S6). Together, these results suggest that *L. monocytogenes* MVs can protect eukaryotic cells against LLO-induced lysis of the plasma membrane, and this may avoid or slow down host cell death during bacterial infection.

### MVs inhibit LLO-induced autophagy by preventing pore formation in eukaryotic cell membrane

**Figure 12:**
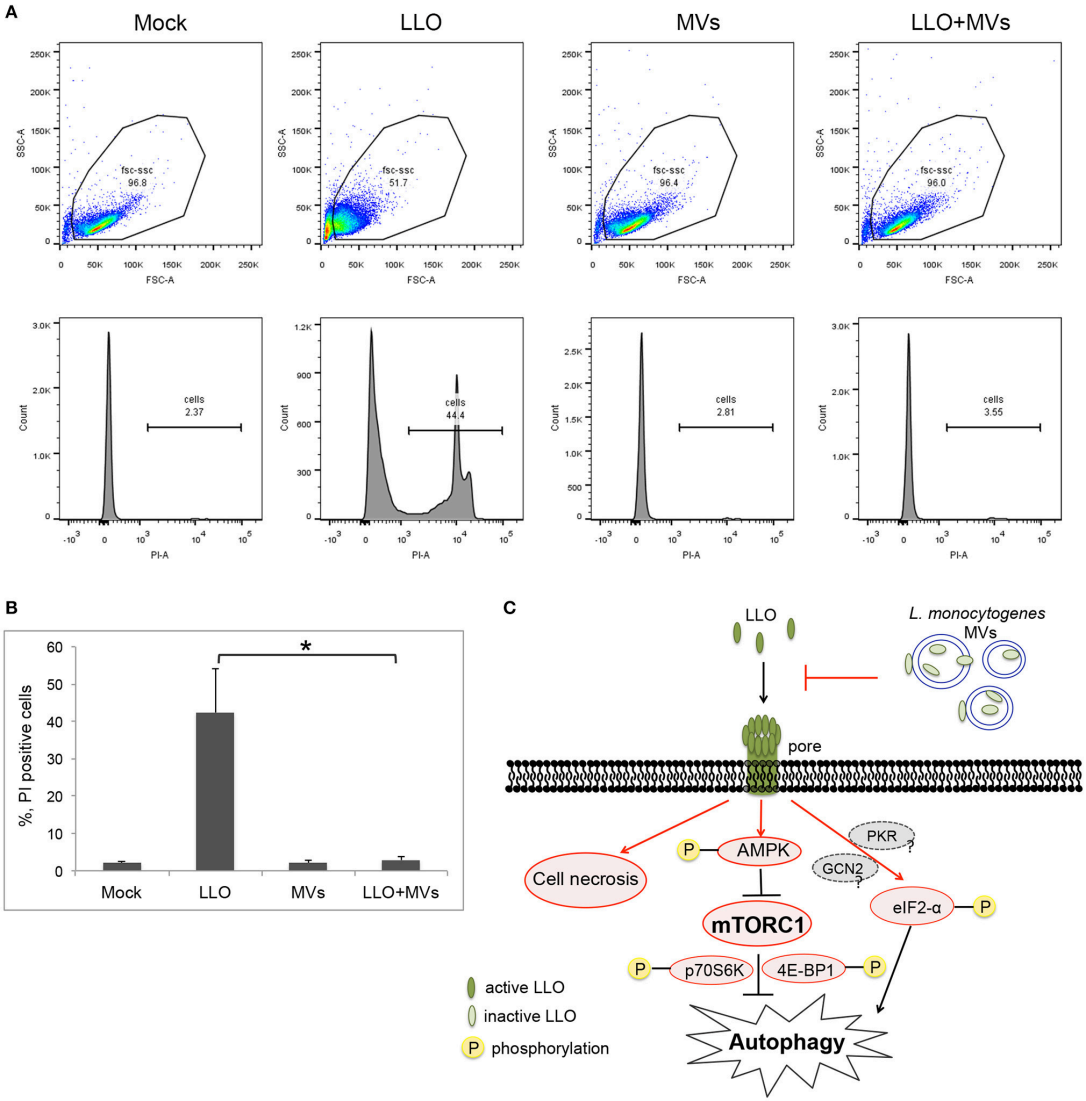
**Analysis of pore formation on the cell membrane using propidium iodide influx assay by flow cytometry analysis. (A,B)** HEK293 cells, pretreated with *L. monocytogenes* MVs (4.5 h) and subsequently with 250 ng/ml LLO for 1 h, were stained with 5 μg/ml propidium iodide (PI) and analyzed by flow cytometry after 15 min incubation with PI. **(A)** Upper panels show cytograms with gating plotting forward scatter (FSC), representing size of cells, against side scatter (SSC) representing granularity. 20,000 events were analyzed by flow cytometry. Lower panels represent histograms showing propidium iodide intake by cells. Mock, treated with 1 × PBS; LLO, treated with pure LLO for 1 h; MVs, treated with MVs for 4.5 h; LLO+MVs, pre-treated with MVs for 4.5 h and treated with LLO for 1 h. **(B)** PI positive cells indicating cell membrane damage were quantified from three independent experiments. ^*^P < 0.05, Student's *t-*test. **(C)** A schematic model for inhibition of LLO-induced pore formation, autophagy and cell necrosis by *L. monocytogenes* MVs. Shapes and arrows in red color represent data from the current study; shapes and arrows in black and dark gray represent data from the pervious studies. Inactive, oxidized LLO is shown in light green color; active, reduced LLO is shown in dark green color.

To further determine whether *L. monocytogenes* MVs prevent pore formation induced by PFTs on the plasma membrane of the host cells, the integrity of plasma membrane was analyzed by monitoring the influx of propidium iodide by flow cytometry after a short treatment with LLO (1 h) at the concentration used for autophagy stimulation (250 ng/ml). Measuring the influx of propidium iodide, a nucleic acid-specific fluorescent marker, is a common method to detect pore formation (Spyr et al., 1995; Fagerlund et al., 2008; Choi H. et al., 2013). When the membrane integrity is impaired, the propidium iodide may diffuse into the cells where it binds to the nucleic acids and can be monitored as fluorescent labeling of the cells. We pre-treated HEK293 cells with MVs and subsequently incubated with 250 ng/ml LLO for 1 h. As expected, LLO induced PI influx, as shown in Figures 12A,B. Strikingly, addition of MVs prior to LLO did not result in any significant PI influx but appeared very similar to mock-treated cells, both in terms of the ability to exclude PI and with respect to the size and granularity of the cells (as analyzed by forward- and side-scatter characteristics).

In order to determine whether MVs can simply bind and sequester toxin, we examined the interaction between LLO and MVs from the *hly* mutant strain. MVs were incubated for 1.5 h with purified LLO and whereas MVs were labeled with PKH26 dye, LLO was detected by immunofluorescence using an anti-LLO antibody. Fluorescence microscopy revealed absence of colocalization between LLO and MVs from the *hly* mutant (Figure S7A). To confirm that there was no sequestration of purified LLO on the surface of MVs from the *hly* mutant strain, we performed immunogold labeling of LLO and electron microscopic analyses. As shown in Figure S7B, there was no association of gold particles on the surfaces of MVs. These results were also confirmed using ELISA (Figure S7C). Our data indicate that MV-mediated autophagy inhibition was not simply due to binding or sequestration of LLO on the surface of MVs.

## Discussion

The majority of studies on bacterial MV production have been focused on Gram-negative bacteria and on the toxicity of MVs to eukaryotic cells or other bacteria (Berleman and Auer, 2013; Thay et al., 2013b). Much remains to be discovered about the prevalence and functions of Gram-positive MVs. Our study reveals a novel protective function of MVs secreted by the intracellular, Gram-positive pathogen *L. monocytogenes*, and a pertinent role of MVs in subverting the autophagic response of the host cell, largely via inhibition of pore formation. There has only been one report previously that has demonstrated MV production by *L. monocytogenes* (Lee et al., 2013). That study described the role of a general stress transcription factor (σ^B^) in stimulating MV production *in vitro* (i.e., in the absence of a host cell), and this was suggested to be important for bacterial survival under harsh environmental conditions (Lee et al., 2013). In the current work, we demonstrated that *L. monocytogenes* produces MVs not only *in vitro*, but also *in vivo* (inside host cells), and intriguingly, we detected a high LLO content in purified MV fractions. Furthermore, SDS solubilization was required to fully extract LLO from isolated MVs, which suggests that LLO, a key virulence factor, is tightly associated with MVs, mainly in the MV lumen and/or embedded in the MV membrane, or partially re-associated on the MV surface. Having one cysteine residue, LLO forms intermolecular disulphide bonds, maintaining the protein in an inactive state. Exposure to a reducing environment destroys the disulphide bonds, and we found that this converted the MV-associated LLO to a hemolytically active state. This bacterial strategy might be needed to regulate LLO levels post-translationally and to keep it inactive with the help of MVs, for example, during persistent infection or during replication inside SLAPs or even during survival outside the host (Ray et al., 2009). Since MVs were unable to bind or sequester LLO *in vitro* (Figure S7), the trapping/embedding of LLO in MVs likely occurs before and/or during the formation of MVs from the bacteria. In our further studies, we focused on understanding the role of *L. monocytogenes* MVs in host-cell interactions.

The relationship between autophagy and bacterial PFTs, including LLO, has been described for several toxin-secreting pathogens (Gutierrez et al., 2007; Saka et al., 2007; Meyer-Morse et al., 2010). Intracellular bacteria, including *Listeria, Shigella* and *Salmonella* species can cause membrane damage, either via the type III secretion system or PFT, leading to metabolic reprogramming and impairment in cellular homeostasis inside the host cells, which in turn might further cause autophagy and host cell death (Kloft et al., 2010). In this study, we observed that autophagy stimulation with two different PFTs (VCC from *V. cholerae* and LLO) was strongly reduced upon pre-treatment of HEK293 cells with *L. monocytogenes* MVs. We found that this occurs due to prevention of pore formation by MVs. Moreover, we demonstrated that MV protection against autophagy is not only effective against PFT-induced autophagy but also against macroautophagy induced by the mTOR-inhibitor Torin1. Interestingly, MVs only counteracted induced autophagy, whereas they did not appear to affect basal autophagy. As shown previously, LLO alone is sufficient to induce autophagy in the absence of infection (Meyer-Morse et al., 2010). In this study, we showed that pure LLO-induced autophagy is related to a strong inhibition of the key autophagy regulator mTORC1. *L. monocytogenes* MVs largely reversed LLO-mediated phosphorylation of AMPK and eIF2α as well as LLO-induced mTORC1 inhibition indicating that MVs might block early signaling of the autophagy machinery. We showed that MVs do not bind and simply sequester purified pore-forming toxin. Rather, it is likely that MVs modify the eukaryotic cell surface and therefore prevent or abolish insertion of pre-pore complexes and/or pore formation by the toxin. Interestingly, MV protein(s) rather than lipids in the *L. monocytogenes* MV structure are likely to be responsible for this inhibition since MV protein, but not lipid extracts, could totally prevent LLO-induced autophagy. However, since MVs were also able to suppress autophagy under conditions where mTOR was permanently inhibited by Torin1, additional mechanisms are likely to be involved. To identify the factors that might be involved in MV-mediated autophagy inhibition, we isolated MVs from the mutant strains of major virulence regulator PrfA, phospholipases PlcA/B or lipoprotein chaperone PrsA2 and tested the autophagy inhibition. However, MVs from mutant strains showed similar protective effects as MVs from wild type (data not shown). Currently, we are trying to identify potential protein component(s) of *L. monocytogenes* MVs that might inhibit autophagy and pore formation. In general, *L. monocytogenes* has developed different alternative strategies to manipulate and escape from the host autophagic defense e.g., by inhibiting cytosolic autophagic targeting using coating complexes on the bacterial cell surface, such as InlK/MVP or ActA/Arp2/3, or by reducing the autophagic flux and PI3P levels through the action of phospholipases C, resulting in stalling of the pre-autophagosomal structures and preventing efficient autophagy targeting of cytosolic bacteria (Dortet et al., 2011; Tattoli et al., 2013). Based on our findings, we suggest that release of MVs from *L. monocytogenes* inside the eukaryotic host cells is an additional bacterial strategy to inhibit a high activity of LLO in part by entrapping and maintaining it in an inactive, oxidized state, and in part by preventing phagosomal and plasma membrane damage and autophagy caused by active, free LLO. Although MVs abolished VCC-induced autophagy as well as that induced by LLO, it remains to be determined to which degree MVs may elicit a general protection against host cell membrane damage.

The relationship between autophagy and different types of cell death is a subject of high interest. The expression of LLO is required for the induction of cell death by *L. monocytogenes*, and LLO can induce cell death as a purified protein (Carrero et al., 2004; Hernández-Flores and Vivanco-Cid, 2015). The outcome of induced cell death by purified LLO is highly dependent on the type of cells, concentration of LLO, and exposure time of the cells to LLO (Carrero et al., 2004; González-Juarbe et al., 2015). Unlike other cholesterol-dependent cytolysins (CDCs), LLO has unique characteristics that can limit its activity to disrupting vacuolar membranes for bacterial escape without killing the infected cell. The low cytotoxic activity of LLO results from several processes including translational repression in the cytosol, pH-dependent denaturation, and degradation by the proteasome (Seveau, 2014). In the current study, we observed that LLO-induced cell lysis, which appeared to occur already within 15 min of cell treatment, and which was unrelated to autophagy, necroptosis and apoptosis, was completely protected by addition of MVs to the cells. To our knowledge, this is the first time that bacterial membrane vesicles have been shown to have powerful protective activity against host cell necrosis initiated by pore forming toxins. These findings allow us to speculate that *in vivo* production of MVs by *L. monocytogenes* might be a relevant strategy for the intracellular survival of bacteria by means of protecting the life of the host cell in cases of very high pore-forming LLO activity.

A common feature for the action of PFTs, including LLO, is disruption of cellular ion homeostasis, causing potassium efflux and calcium influx upon plasma membrane damage. Transcellular ion dysregulation can further lead to activation of an intracellular energy sensor, AMP-activated protein kinase (AMPK), to autophagy induction and rapid necrosis, apoptosis or necroptosis depending on the cell type, dose and exposure of PFT (Essmann et al., 2003; Kennedy et al., 2009; Mathieu, 2015). As a survival strategy, cells can repair membrane injuries and seal the pores. Interestingly, the pore-sealing process has a different timescale depending on pore size: larger pores made by LLO or SLO are sealed within minutes, while smaller pores made by aerolysin are sealed within hours. Plasma membrane pores formed by SLO are endocytosed via a calcium-dependent endocytic mechanism, and subsequently the pore-endosome complexes are ubiquitinated, leading to lysosomal degradation (Idone et al., 2008; Gonzalez et al., 2011; Corrotte et al., 2012; Hamon et al., 2012; Los et al., 2013). Pore-sealing endocytosis is reminiscent of LLO-induced bacterial internalization although these two processes are distinct. Considering the highly protective effect of MVs against membrane-damaging actions of LLO, we suggest that the protective functions of *L. monocytogenes* MVs could result from pore sealing or prevention of new pore formations in the host plasma membrane as indicated in the schematic summary of how MVs may block LLO-induced autophagy (Figure 12C). The mechanism of Torin1-induced autophagy inhibition by MVs might be different from pore forming toxin induced autophagy inhibition since the effect of Torin-1 was known to be direct on mTOR. Elucidating the detailed mechanism(s) of pore forming toxin or Torin-1 induced autophagy inhibition by MVs and the roles for *L. monocytogenes* MVs during infection remain challenging but of great scientific interest.

*L. monocytogenes* MVs might use different intracellular routes during infection. As we demonstrated, MVs can be endocytosed by non-phagocytic cells independently of bacteria and shortly entrapped inside early endosomes, further transported to lysosomes where they accumulate. On the other hand, some bacteria entrapped within the phagosome may release MVs, which could down-regulate the autophagic response induced by host cell membrane damage. Consequently, different intracellular routes of MVs can likely lead to different outcomes of infection. In this study, we observed a two-fold increase in intracellular levels of *L. monocytogenes* when MEFs were pre-treated with MVs, highlighting the importance of MVs secretion during intracellular infection. Further, studies would be necessary to decipher the mechanism(s) by which MVs can enhance intracellular survival of bacteria.

Eukaryotic cells can use autophagy as a basal housekeeping function to avoid accumulation of dysfunctional molecules and organelles, but also for the removal of invading pathogens. Certain pathogens have evolved strategies to evade autophagy (Thurston et al., 2012; Benjamin et al., 2013; Choy and Roy, 2013). The mechanisms by which bacteria evade autophagy remain mostly unclear and are currently the topic of intense investigation. Recently, it was demonstrated that the *Salmonella* virulence plasmid harboring *spv* genes enhances intracellular bacterial survival by inhibiting autophagy in the host cells. It occurs through interference with the initial stage of autophagy by depolymerization of the actin cytoskeleton (Chu et al., 2016). Based on our findings, we suggest that release of MVs from *L. monocytogenes* inside the eukaryotic host cells might be a strategy to inhibit the autophagy system, which becomes activated upon membrane damage caused by active LLO, and moreover, protect cells against the lytic action of LLO. Taken together, our study broadens our knowledge of Gram-positive bacterial MVs and of *L. monocytogenes* pathogenesis. Autophagy is implicated in a whole range of serious human pathologies including cancer, neurodegenerative diseases, diabetes, cardiovascular diseases, inflammatory bowel disease, and diseases caused by human immunodeficiency virus (HIV) and hepatitis C virus (HCV; Choi A. M. et al., 2013; Jiang and Mizushima, 2014). There is, thus, a great clinical interest in therapeutically modulating autophagy. Our findings that *L. monocytogenes* MVs, unlike MVs from other bacterial species, suppress autophagy and are easily taken up by cells introduce a new possible avenue in the development of therapeutic strategies to target autophagy. From another perspective, cell membrane damage is the cause of many diseases, such as, for example, multiple sclerosis, cystic fibrosis, and Alzheimer disease (Goldberg and Riordan, 1986; Lukiw, 2013). In conclusion, a better understanding of the mechanism(s) by which MVs protect from pore formation and damage of the host plasma membrane and of their effect on autophagy will broaden our understanding of intracellular infection and identify novel putative therapeutic targets.

## Authors contributions

Design of the study: SV, ML, PS, LN, MF, RL, NE, JJ, and SW. Experimental part: SV, ML, PS, LN, and MF. Data analysis and interpretation: SV, ML, PS, LN, MF, RL, NE, JJ, and SW. Writing the manuscript: SV, ML, PS, LN, MF, RL, NE, JJ, and SW. Final approval: SV, ML, PS, LN, MF, RL, NE, JJ, and SW.

## Funding

This work was performed within the Umeå Centre for Microbial Research (UCMR) Linnaeus Programme and was supported by grants from the Swedish Research Council (SW: 2013-2392 (VR-M), 2014-4401 (VR-NT), Cancerfonden (CAN2014/831); JJ: 621-2012-2451 (VR) and the Faculty of Medicine at Umeå University.

### Conflict of interest statement

The authors declare that the research was conducted in the absence of any commercial or financial relationships that could be construed as a potential conflict of interest.

## References

[B1] Ahmad M. Shi Y. (2000). TRAIL-induced apoptosis of thyroid cancer cells: potential for therapeutic intervention. Oncogene 19, 3363–3371. 10.1038/sj.onc.1203679 10918593

[B2] Aroian R. van der Goot F. G. (2007). Pore-forming toxins and cellular non-immune defenses (CNIDs). Curr. Opin. Microbiol. 10, 57–61. 10.1016/j.mib.2006.12.008 17234446

[B3] Ayala G. Torres L. Espinosa M. Fierros-Zarate G. Maldonado V. Meléndez-Zajgla J. (2006). External membrane vesicles from *Helicobacter pylori* induce apoptosis in gastric epithelial cells. FEMS Microbiol. Lett. 260, 178–185. 10.1111/j.1574-6968.2006.00305.x 16842342

[B4] Balsalobre C. Silván J. M. Berglund S. Mizunoe Y. Uhlin B. E. Wai S. N. (2006). Release of the type I secreted alpha-haemolysin via outer membrane vesicles from *Escherichia coli*. Mol. Microbiol. 59, 99–112. 10.1111/j.1365-2958.2005.04938.x 16359321

[B5] Benjamin J. L. Sumpter R. Jr. Levine B. Hooper L. V. (2013). Intestinal epithelial autophagy is essential for host defense against invasive bacteria. Cell Host Microbe 13, 723–734. 10.1016/j.chom.2013.05.004 23768496 PMC3755484

[B6] Berleman J. Auer M. (2013). The role of bacterial outer membrane vesicles for intra- and interspecies delivery. Environ. Microbiol. 15, 347–354. 10.1111/1462-2920.12048 23227894

[B7] Bielig H. Rompikuntal P. K. Dongre M. Zurek B. Lindmark B. Ramstedt M.. (2011). NOD-like receptor activation by outer membrane vesicles from *Vibrio cholerae* non-O1 non-O139 strains is modulated by the quorum-sensing regulator HapR. Infect. Immun. 79, 1418–1427. 10.1128/IAI.00754-10 21263023 PMC3067550

[B8] Birmingham C. L. Canadien V. Gouin E. Troy E. B. Yoshimori T. Cossart P.. (2007). *Listeria monocytogenes* evades killing by autophagy during colonization of host cells. Autophagy 3, 442–451. 10.4161/auto.4450 17568179

[B9] Birmingham C. L. Canadien V. Kaniuk N. A. Steinberg B. E. Higgins D. E. Brumell J. H. (2008a). Listeriolysin O allows *Listeria monocytogenes* replication in macrophage vacuoles. Nature 451, 350–354. 10.1038/nature06479 18202661

[B10] Birmingham C. L. Higgins D. E. Brumell J. H. (2008b). Avoiding death by autophagy: interactions of *Listeria monocytogenes* with the macrophage autophagy system. Autophagy 4, 368–371. 10.4161/auto.5594 18216493

[B11] Bolte S. Cordelières F. P. (2006). A guided tour into subcellular colocalization analysis in light microscopy. J. Microsc. 224, 213–232. 10.1111/j.1365-2818.2006.01706.x 17210054

[B12] Campoy E. Colombo M. I. (2009). Autophagy in intracellular bacterial infection. Biochim. Biophys. Acta 1793, 1465–1477. 10.1016/j.bbamcr.2009.03.003 19303905

[B13] Carrero J. A. Calderon B. Unanue E. R. (2004). Listeriolysin O from *Listeria monocytogenes* is a lymphocyte apoptogenic molecule. J. Immunol. 172, 4866–4874. 10.4049/jimmunol.172.8.4866 15067065

[B14] Carrero J. A. Vivanco-Cid H. Unanue E. R. (2008). Granzymes drive a rapid listeriolysin O-induced T cell apoptosis. J. Immunol. 181, 1365–1374. 10.4049/jimmunol.181.2.1365 18606691 PMC2562634

[B15] Choi A. M. Ryter S. W. Levine B. (2013). Autophagy in human health and disease. N. Engl. J. Med. 368, 1845–1846. 10.1056/NEJMc1303158 23656658

[B16] Choi H. Hwang J. S. Lee D. G. (2013). Antifungal effect and pore-forming action of lactoferricin B like peptide derived from centipede *Scolopendra subspinipes mutilans*. Biochim. Biophys. Acta 1828, 2745–2750. 10.1016/j.bbamem.2013.07.021 23896552

[B17] Choy A. Roy C. R. (2013). Autophagy and bacterial infection: an evolving arms race. Trends Microbiol. 21, 451–456. 10.1016/j.tim.2013.06.009 23880062 PMC3839292

[B18] Chu Y. Gao S. Wang T. Yan J. Xu G. Li Y.. (2016). A novel contribution of spvB to pathogenesis of Salmonella Typhimurium by inhibiting autophagy in host cells. Oncotarget 7, 8295–8309. 10.18632/oncotarget.6989 26811498 PMC4884993

[B19] Corrotte M. Fernandes M. C. Tam C. Andrews N. W. (2012). Toxin pores endocytosed during plasma membrane repair traffic into the lumen of MVBs for degradation. Traffic 13, 483–494. 10.1111/j.1600-0854.2011.01323.x 22212686 PMC3356596

[B20] Dal Peraro M. van der Goot F. G. (2016). Pore-forming toxins: ancient, but never really out of fashion. Nat. Rev. Microbiol. 14, 77–92. 10.1038/nrmicro.2015.3 26639780

[B21] Dortet L. Mostowy S. Cossart P. (2012). Listeria and autophagy escape: involvement of InlK, an internalin-like protein. Autophagy 8, 132–134. 10.4161/auto.8.1.18218 22082958 PMC3335995

[B22] Dortet L. Mostowy S. Samba-Louaka A. Gouin E. Nahori M. A. Wiemer E. A. C.. (2011). Recruitment of the major vault protein by InlK: a *Listeria monocytogenes* strategy to avoid autophagy. PLoS Pathog. 7:e1002168. 10.1371/annotation/a70544fc-6d8b-4549-921a-9e86557b0ffc 21829365 PMC3150275

[B23] Duperthuy M. Sjöström A. E. Sabharwal D. Damghani F. Uhlin B. E. Wai S. N. (2013). Role of the *Vibrio cholerae* matrix protein Bap1 in cross-resistance to antimicrobial peptides. PLoS Pathog. 9:e1003620. 10.1371/journal.ppat.1003620 24098113 PMC3789753

[B24] Elluri S. Enow C. Vdovikova S. Rompikuntal P. K. Dongre M. Carlsson S.. (2014). Outer membrane vesicles mediate transport of biologically active *Vibrio cholerae* cytolysin (VCC) from V. cholerae strains. PLoS ONE 9:e106731. 10.1371/journal.pone.0106731 25187967 PMC4154730

[B25] Essmann F. Bantel H. Totzke G. Engels I. H. Sinha B. Schulze-Osthoff K.. (2003). *Staphylococcus aureus* alpha-toxin-induced cell death: predominant necrosis despite apoptotic caspase activation. Cell Death Differ. 10, 1260–1272. 10.1038/sj.cdd.4401301 12894214

[B26] Fagerlund A. Lindbäck T. Storset A. K. Granum P. E. Hardy S. P. (2008). *Bacillus cereus* Nhe is a pore-forming toxin with structural and functional properties similar to the ClyA (HlyE, SheA) family of haemolysins, able to induce osmotic lysis in epithelia. Microbiology 154(Pt 3), 693–704. 10.1099/mic.0.2007/014134-0 18310016

[B27] Farber J. M. Peterkin P. I. (1991). *Listeria monocytogenes*, a food-borne pathogen. Microbiol. Rev. 55, 476–511. 1943998 10.1128/mr.55.3.476-511.1991 PMC372831

[B28] Goldberg D. M. Riordan J. R. (1986). Role of membranes in disease. Clin. Physiol. Biochem. 4, 305–336. 3022980

[B29] González-Juarbe N. Gilley R. P. Hinojosa C. A. Bradley K. M. Kamei A. Gao G.. (2015). Pore-forming toxins induce macrophage necroptosis during acute bacterial pneumonia. PLoS Pathog. 11:e1005337. 10.1371/journal.ppat.1005337 26659062 PMC4676650

[B30] Gonzalez M. R. Bischofberger M. Frêche B. Ho S. Parton R. G. van der Goot F. G. (2011). Pore-forming toxins induce multiple cellular responses promoting survival. Cell. Microbiol. 13, 1026–1043. 10.1111/j.1462-5822.2011.01600.x 21518219

[B31] Gurung M. Moon D. C. Choi C. W. Lee J. H. Bae Y. C. Kim J.. (2011). *Staphylococcus aureus* produces membrane-derived vesicles that induce host cell death. PLoS ONE 6:e27958. 10.1371/journal.pone.0027958 22114730 PMC3218073

[B32] Gutierrez M. G. Saka H. A. Chinen I. Zoppino F. C. Yoshimori T. Bocco J. L.. (2007). Protective role of autophagy against *Vibrio cholerae* cytolysin, a pore-forming toxin from *V*. cholerae. Proc. Natl. Acad. Sci. U.S.A. 104, 1829–1834. 10.1073/pnas.0601437104 17267617 PMC1794277

[B33] Haas B. Grenier D. (2015). Isolation, Characterization and biological properties of membrane vesicles produced by the swine pathogen *Streptococcus suis*. PLoS ONE 10:e0130528. 10.1371/journal.pone.0130528 26110524 PMC4482388

[B34] Hamon M. A. Batsché E. Régnault B. Tham T. N. Seveau S. Muchardt C.. (2007). Histone modifications induced by a family of bacterial toxins. Proc. Natl. Acad. Sci. U.S.A. 104, 13467–13472. 10.1073/pnas.0702729104 17675409 PMC1948930

[B35] Hamon M. A. Ribet D. Stavru F. Cossart P. (2012). Listeriolysin O: the Swiss army knife of *Listeria*. Trends Microbiol. 20, 360–368. 10.1016/j.tim.2012.04.006 22652164

[B36] Han W. Li L. Qiu S. Lu Q. Pan Q. Gu Y.. (2007). Shikonin circumvents cancer drug resistance by induction of a necroptotic death. Mol. Cancer Ther. 6, 1641–1649. 10.1158/1535-7163.MCT-06-0511 17513612

[B37] He C. Klionsky D. J. (2009). Regulation mechanisms and signaling pathways of autophagy. Annu. Rev. Genet. 43, 67–93. 10.1146/annurev-genet-102808-114910 19653858 PMC2831538

[B38] Hernández-Flores K. G. Vivanco-Cid H. (2015). Biological effects of listeriolysin O: implications for vaccination. Biomed Res. Int. 2015:360741. 10.1155/2015/360741 25874208 PMC4385656

[B39] Huang J. Brumell J. H. (2014). Bacteria-autophagy interplay: a battle for survival. Nat. Rev. Microbiol. 12, 101–114. 10.1038/nrmicro3160 24384599 PMC7097477

[B40] Iacovache I. van der Goot F. G. Pernot L. (2008). Pore formation: an ancient yet complex form of attack. Biochim. Biophys. Acta 1778, 1611–1623. 10.1016/j.bbamem.2008.01.026 18298943

[B41] Idone V. Tam C. Goss J. W. Toomre D. Pypaert M. Andrews N. W. (2008). Repair of injured plasma membrane by rapid Ca2+-dependent endocytosis. J. Cell Biol. 180, 905–914. 10.1083/jcb.200708010 18316410 PMC2265401

[B42] Jiang P. Mizushima N. (2014). Autophagy and human diseases. Cell Res. 24, 69–79. 10.1038/cr.2013.161 24323045 PMC3879707

[B43] Jin J. S. Kwon S. O. Moon D. C. Gurung M. Lee J. H. Kim S. I.. (2011). *Acinetobacter baumannii* secretes cytotoxic outer membrane protein A via outer membrane vesicles. PLoS ONE 6:e17027. 10.1371/journal.pone.0017027 21386968 PMC3046175

[B44] Kaushik S. Cuervo A. M. (2012). Chaperones in autophagy. Pharmacol. Res. 66, 484–493. 10.1016/j.phrs.2012.10.002 23059540 PMC3502706

[B45] Kayal S. Charbit A. (2006). Listeriolysin O: a key protein of *Listeria monocytogenes* with multiple functions. FEMS Microbiol. Rev. 30, 514–529. 10.1111/j.1574-6976.2006.00021.x 16774585

[B46] Kayal S. Lilienbaum A. Join-Lambert O. Li X. Israël A. Berche P. (2002). Listeriolysin O secreted by *Listeria monocytogenes* induces NF-kappaB signalling by activating the IkappaB kinase complex. Mol. Microbiol. 44, 1407–1419. 10.1046/j.1365-2958.2002.02973.x 12028384

[B47] Kennedy C. L. Smith D. J. Lyras D. Chakravorty A. Rood J. I. (2009). Programmed cellular necrosis mediated by the pore-forming alpha-toxin from *Clostridium septicum*. PLoS Pathog. 5:e1000516. 10.1371/journal.ppat.1000516 19609357 PMC2705182

[B48] Keyel P. A. Roth R. Yokoyama W. M. Heuser J. E. Salter R. D. (2013). Reduction of streptolysin O (SLO) pore-forming activity enhances inflammasome activation. Toxins (Basel) 5, 1105–1118. 10.3390/toxins5061105 23744055 PMC3717772

[B49] Khilwani B. Chattopadhyay K. (2015). Signaling beyond punching holes: modulation of cellular responses by *Vibrio cholerae* Cytolysin. Toxins (Basel) 7, 3344–3358. 10.3390/toxins7083344 26308054 PMC4549754

[B50] Klionsky D. J. Abdelmohsen K. Abe A. Abedin M. J. Abeliovich H. Acevedo Arozena A.. (2016). Guidelines for the use and interpretation of assays for monitoring autophagy (3rd edition). Autophagy 12, 1–222. 10.1080/15548627.2015.1100356 26799652 PMC4835977

[B51] Kloft N. Neukirch C. Bobkiewicz W. Veerachato G. Busch T. von Hoven G.. (2010). Pro-autophagic signal induction by bacterial pore-forming toxins. Med. Microbiol. Immunol. 199, 299–309. 10.1007/s00430-010-0163-0 20454906 PMC2955911

[B52] Konings W. N. Freese E. (1972). Amino acid transport in membrane vesicles of *Bacillus subtilis*. J. Biol. Chem. 247, 2408–2418. 4401701

[B53] Kulp A. Kuehn M. J. (2010). Biological functions and biogenesis of secreted bacterial outer membrane vesicles. Annu. Rev. Microbiol. 64, 163–184. 10.1146/annurev.micro.091208.073413 20825345 PMC3525469

[B54] Laemmli U. K. (1970). Cleavage of structural proteins during the assembly of the head of bacteriophage T4. Nature 227, 680–685. 10.1038/227680a0 5432063

[B55] Lam G. Y. Cemma M. Muise A. M. Higgins D. E. Brumell J. H. (2013). Host and bacterial factors that regulate LC3 recruitment to *Listeria monocytogenes* during the early stages of macrophage infection. Autophagy 9, 985–995. 10.4161/auto.24406 23584039 PMC3722333

[B56] Lam G. Y. Czuczman M. A. Higgins D. E. Brumell J. H. (2012). Interactions of *Listeria monocytogenes* with the autophagy system of host cells. Adv. Immunol. 113, 7–18. 10.1016/B978-0-12-394590-7.00008-7 22244576

[B57] Laplante M. Sabatini D. M. (2009). mTOR signaling at a glance. J Cell Sci 122(Pt 20), 3589–3594. 10.1242/jcs.051011 19812304 PMC2758797

[B58] LaRocca T. J. Stivison E. A. Hod E. A. Spitalnik S. L. Cowan P. J. Randis T. M.. (2014). Human-specific bacterial pore-forming toxins induce programmed necrosis in erythrocytes. MBio 5, e01251–e01214. 10.1128/mBio.01251-14 25161188 PMC4173772

[B59] Lee J. Giordano S. Zhang J. (2012). Autophagy, mitochondria and oxidative stress: cross-talk and redox signalling. Biochem. J. 441, 523–540. 10.1042/BJ20111451 22187934 PMC3258656

[B60] Lee J. H. Choi C. W. Lee T. Kim S. I. Lee J. C. Shin J. H. (2013). Transcription factor σB plays an important role in the production of extracellular membrane-derived vesicles in *Listeria monocytogenes*. PLoS ONE 8:e73196. 10.1371/journal.pone.0073196 23977379 PMC3748028

[B61] Lindmark B. Rompikuntal P. K. Vaitkevicius K. Song T. Mizunoe Y. Uhlin B. E.. (2009). Outer membrane vesicle-mediated release of cytolethal distending toxin (CDT) from *Campylobacter jejuni*. BMC Microbiol. 9:220. 10.1186/1471-2180-9-220 19835618 PMC2770062

[B62] Los F. C. Randis T. M. Aroian R. V. Ratner A. J. (2013). Role of pore-forming toxins in bacterial infectious diseases. Microbiol. Mol. Biol. Rev. 77, 173–207. 10.1128/MMBR.00052-12 23699254 PMC3668673

[B63] Lukiw W. J. (2013). Alzheimer's disease (AD) as a disorder of the plasma membrane. Front. Physiol. 4:24. 10.3389/fphys.2013.00024 23424582 PMC3573332

[B64] Mathieu J. (2015). Interactions between autophagy and bacterial toxins: targets for therapy? Toxins (Basel) 7, 2918–2958. 10.3390/toxins7082918 26248079 PMC4549733

[B65] Meyer-Morse N. Robbins J. R. Rae C. S. Mochegova S. N. Swanson M. S. Zhao Z.. (2010). Listeriolysin O is necessary and sufficient to induce autophagy during *Listeria monocytogenes* infection. PLoS ONE 5:e8610. 10.1371/journal.pone.0008610 20062534 PMC2797616

[B66] Nakagawa I. Amano A. Mizushima N. Yamamoto A. Yamaguchi H. Kamimoto T.. (2004). Autophagy defends cells against invading group A Streptococcus. Science 306, 1037–1040. 10.1126/science.1103966 15528445

[B67] Ou G. Rompikuntal P. K. Bitar A. Lindmark B. Vaitkevicius K. Wai S. N.. (2009). *Vibrio cholerae* cytolysin causes an inflammatory response in human intestinal epithelial cells that is modulated by the PrtV protease. PLoS ONE 4:e7806. 10.1371/journal.pone.0007806 19907657 PMC2771358

[B68] Palmer M. (2001). The family of thiol-activated, cholesterol-binding cytolysins. Toxicon 39, 1681–1689. 10.1016/S0041-0101(01)00155-6 11595631

[B69] Pasquier B. (2015). SAR405, a PIK3C3/Vps34 inhibitor that prevents autophagy and synergizes with MTOR inhibition in tumor cells. Autophagy 11, 725–726. 10.1080/15548627.2015.1033601 25905679 PMC4502822

[B70] Portnoy D. A. Auerbuch V. Glomski I. J. (2002). The cell biology of *Listeria monocytogenes* infection: the intersection of bacterial pathogenesis and cell-mediated immunity. J. Cell Biol. 158, 409–414. 10.1083/jcb.200205009 12163465 PMC2173830

[B71] Py B. F. Lipinski M. M. Yuan J. (2007). Autophagy limits *Listeria monocytogenes* intracellular growth in the early phase of primary infection. Autophagy 3, 117–125. 10.4161/auto.3618 17204850

[B72] Ray K. Marteyn B. Sansonetti P. J. Tang C. M. (2009). Life on the inside: the intracellular lifestyle of cytosolic bacteria. Nat. Rev. Microbiol. 7, 333–340. 10.1038/nrmicro2112 19369949

[B73] Rivera J. Cordero R. J. Nakouzi A. S. Frases S. Nicola A. Casadevall A. (2010). *Bacillus anthracis* produces membrane-derived vesicles containing biologically active toxins. Proc. Natl. Acad. Sci. U.S.A. 107, 19002–19007. 10.1073/pnas.1008843107 20956325 PMC2973860

[B74] Rompikuntal P. K. Vdovikova S. Duperthuy M. Johnson T. L. Åhlund M. Lundmark R.. (2015). Outer membrane vesicle-mediated export of processed PrtV Protease from *Vibrio cholerae*. PLoS ONE 10:e0134098. 10.1371/journal.pone.0134098 26222047 PMC4519245

[B75] Saka H. A. Gutiérrez M. G. Bocco J. L. Colombo M. I. (2007). The autophagic pathway: a cell survival strategy against the bacterial pore-forming toxin *Vibrio cholerae* cytolysin. Autophagy 3, 363–365. 10.4161/auto.4159 17404497

[B76] Schnupf P. Portnoy D. A. (2007). Listeriolysin O: a phagosome-specific lysin. Microbes Infect. 9, 1176–1187. 10.1016/j.micinf.2007.05.005 17720603

[B77] Seglen P. O. Luhr M. Mills I. G. Sætre F. Szalai P. Engedal N. (2015). Macroautophagic cargo sequestration assays. Methods 75, 25–36. 10.1016/j.ymeth.2014.12.021 25576638

[B78] Seveau S. (2014). Multifaceted activity of listeriolysin O, the cholesterol-dependent cytolysin of *Listeria monocytogenes*. Subcell. Biochem. 80, 161–195. 10.1007/978-94-017-8881-6_9 24798012 PMC4266574

[B79] Shibutani S. T. Tamotsu Y. (2013). A current perspective of autophagosome biogenesis. Cell Res. 24, 58–68. 10.1038/cr.2013.159 24296784 PMC3879706

[B80] Shaughnessy L. M. Hoppe A. D. Christensen K. A. Swanson J. A. (2006). Membrane perforations inhibit lysosome fusion by altering pH and calcium in *Listeria monocytogenes* vacuoles. Cell. Microbiol. 8, 781–792. 10.1111/j.1462-5822.2005.00665.x 16611227 PMC1435990

[B81] Shibutani S. T. Yoshimori T. (2014). Autophagosome formation in response to intracellular bacterial invasion. Cell. Microbiol. 16, 1619–1626. 10.1111/cmi.12357 25180443

[B82] Smith G. A. Marquis H. Jones S. Johnston N. C. Portnoy D. A. Goldfine H. (1995). The two distinct phospholipases C of *Listeria monocytogenes* have overlapping roles in escape from a vacuole and cell-to-cell spread. Infect. Immun. 63, 4231–4237. 7591052 10.1128/iai.63.11.4231-4237.1995 PMC173601

[B83] Spyr C. A. Käsermann F. Kempf C. (1995). Identification of the pore forming element of Semliki Forest virus spikes. FEBS Lett. 375, 134–136. 10.1016/0014-5793(95)01197-M 7498462

[B84] Szalai P. Hagen L. K. Sætre F. Luhr M. Sponheim M. Øverbye A.. (2015). Autophagic bulk sequestration of cytosolic cargo is independent of LC3, but requires GABARAPs. Exp. Cell Res. 333, 21–38. 10.1016/j.yexcr.2015.02.003 25684710

[B85] Tattoli I. Sorbara M. T. Yang C. Tooze S. A. Philpott D. J. Girardin S. E. (2013). *Listeria* phospholipases subvert host autophagic defenses by stalling pre-autophagosomal structures. EMBO J. 32, 3066–3078. 10.1038/emboj.2013.234 24162724 PMC3844955

[B86] Thay B. Wai S. N. Oscarsson J. (2013a). *Staphylococcus aureus* alpha-toxin-dependent induction of host cell death by membrane-derived vesicles. PLoS ONE 8:e54661. 10.1371/journal.pone.0054661 23382935 PMC3561366

[B87] Thay B. Wai S. N. Oscarsson J. (2013b). *Staphylococcus aureus* α-toxin-dependent induction of host cell death by membrane-derived vesicles. PLoS ONE 8:e54661. 10.1371/journal.pone.0054661 23382935 PMC3561366

[B88] Thurston T. L. Wandel M. P. von Muhlinen N. Foeglein A. Randow F. (2012). Galectin 8 targets damaged vesicles for autophagy to defend cells against bacterial invasion. Nature 482, 414–418. 10.1038/nature10744 22246324 PMC3343631

[B89] Vázquez-Boland J. A. Kuhn M. Berche P. Chakraborty T. Domínguez-Bernal G. Goebel W.. (2001). *Listeria* pathogenesis and molecular virulence determinants. Clin. Microbiol. Rev. 14, 584–640. 10.1128/CMR.14.3.584-640.2001 11432815 PMC88991

[B90] von Hoven G. Kloft N. Neukirch C. Ebinger S. Bobkiewicz W. Weis S.. (2012). Modulation of translation and induction of autophagy by bacterial exoproducts. Med. Microbiol. Immunol. 201, 409–418. 10.1007/s00430-012-0271-0 22991039 PMC3470817

[B91] Wai S. N. Lindmark B. Söderblom T. Takade A. Westermark M. Oscarsson J.. (2003). Vesicle-mediated export and assembly of pore-forming oligomers of the enterobacterial ClyA cytotoxin. Cell 115, 25–35. 10.1016/S0092-8674(03)00754-2 14532000

[B92] Westbrook D. G. Bhunia A. K. (2000). Dithiothreitol enhances *Listeria monocytogenes* mediated cell cytotoxicity. Microbiol. Immunol. 44, 431–438. 10.1111/j.1348-0421.2000.tb02517.x 10941925

[B93] Witte C. E. Archer K. A. Rae C. S. Sauer J. D. Woodward J. J. Portnoy D. A. (2012). Innate immune pathways triggered by *Listeria monocytogenes* and their role in the induction of cell-mediated immunity. Adv. Immunol. 113, 135–156. 10.1016/B978-0-12-394590-7.00002-6 22244582

[B94] Yoshikawa Y. Ogawa M. Hain T. Yoshida M. Fukumatsu M. Kim M.. (2009). *Listeria monocytogenes* ActA-mediated escape from autophagic recognition. Nat. Cell Biol. 11, 1233–1240. 10.1038/ncb1967 19749745

[B95] Yoshimori T. Yamamoto A. Moriyama Y. Futai M. Tashiro Y. (1991). Bafilomycin A1, a specific inhibitor of vacuolar-type H(+)-ATPase, inhibits acidification and protein degradation in lysosomes of cultured cells. J. Biol. Chem. 266, 17707–17712. 1832676

[B96] Zhao Z. Xu Y. (2010). An extremely simple method for extraction of lysophospholipids and phospholipids from blood samples. J. Lipid Res. 51, 652–659. 10.1194/jlr.D001503 19783525 PMC2817595

