# Supplementary material for: A Novel Role of Listeria monocytogenes Membrane Vesicles in Inhibition of Autophagy and Cell Death
Source: Front Cell Infect Microbiol. 2017 May 3;7:154. doi: 10.3389/fcimb.2017.00154 (PMC5413512; doi:10.3389/fcimb.2017.00154)
Supplement: Supplementary file 1 [file Presentation1.pdf]

Supplementary Figure Legends

Fig. S1. *L. monocytogenes* MVs do not colocalize with LC3-phagosomes in HeLa and HEK293 cells. HeLa **(A)** and HEK293 cells **(B)** were treated with 100 μg of PKH26 pre-labeled MVs (red) for 1, 3 and 6 hours, and stained with anti-LC3 antibody in case of HeLa cells (green), GFP-LC3 (green) fluorescence in case of HEK293 cells and DAPI in both cases (blue). Absence of colocalization is indicated with yellow arrowheads. Left panels show an overview of the cell and the square marks the magnified area on the right image. MVs and LC3/GFP-LC3 are shown individually and as a merged image of all three channels. Scale bar: 10 µm (overview) and 2 µm (zoom). **(C)** Table showing average values and standard deviation of Pearson's correlation coefficient for colocalization between MVs and endosomes after 1 and 3 hours or lysosomes after 1, 3 or 6 hours, in HeLa and HEK293 cells.

Fig. S2. Estimation of MV-associated LLO concentration. Purified recombinant LLO (500, 250, 125, 62, 31 and 16 ng) and crude vesicle extracts (10 μl equal to 5 μg, 1:2, 1:4, 1:8 and 1:16 dilutions) were analyzed by immunoblotting using polyclonal anti-LLO antiserum. Band density (arbitrary units) was correlated to LLO amount loaded per well (ng) with the help of the standard curve. Estimated concentration of LLO associated with vesicles is 23 μg/ml.

Fig. S3. Confocal microscopic analysis showing absence of autophagy stimulation by *L. monocytogenes* EGDe MVs. (**A**) HEK293-GFP-LC3 cells were mock-treated (1xPBS) or (**B**) treated with EGDe MVs (200 μg), **(C**) with EGDeΔ*hly* MVs or (**D**) pre-treated with EGDeΔ*hly* MVs before LLO addition. Absence of GFP-LC3 puncta indicates the absence of the autophagy stimulation by MVs or MV-associated LLO. Scale bars: 10 μm.

Fig. S4. Bacterial viability and MVs activity after 16 hours of growth compared to 40 hours. EGDe MVs were isolated from bacterial cultures grown for 16h, concentrated and used in the same concentration as MVs isolated after 40 h of growth (200 μg) to treat the HEK293 cells. (**A)** mock-treated cells, (**B)** LLO-induced autophagy, (**C)** cells pre-treated with MVs isolated from earlier time point and LLO-treated after. LC3 conversion and actin levels are presented in (**D**), LC3-II to actin ratio is quantified and normalised. (**E)** Bacterial viability is quantified and compared after 16 and 40 hours, showing no significant difference.

Fig. S5.

Vibrio cholerae* cytolysin (VCC) and LLO are not degraded by *L. monocytogenes* MVs *in vitro. (**A**) Purified LLO and EGDeΔ*hly* MVs were incubated together for 1.5 hours *in vitro* and LLO was further analyzed by immunoblotting using polyclonal anti-LLO antibody; (**B**) MVs from both bacteria *L. monocytogenes* and *V. cholerae* were incubated together for 6 hours *in vitro* and VCC was further analyzed by immunoblotting using polyclonal anti-VCC antibody.

Fig. S6.

Verification of cell death inhibitors. The anti-necroptotic effect of Nec-1 and anti-apoptotic effect of Z-VAD-FMK were verified by inducing necroptosis with shikonin (4 μM) and apoptosis with TRAIL (160 ng/ml) in combination with cycloheximide

(CHX, 20 ng/ml), respectively, for 4 hours, and further 1 h incubation with Nec-1 and Z-VAD-FMK. Images were acquired using live-cell imaging. Scale bar: 100 µm.

Fig. S7.

Verification of binding activity and sequestration of PFTs by *L. monocytogenes* MVs *in vitro*. EGDeΔ*hly* MVs and purified LLO were incubated *in vitro*, ultracentrifuged and binding activity was analyzed using electron microscopy and anti-LLO immunogold labeling. **(A)** Immunofluorescence microscopy using anti-LLO polyclonal antibody. For fluorescence microscopy analysis MVs were stained using PKH26 Red Fluorescent dye (red; red arrows) and LLO was detected using anti-LLO FITC-labelled anti-rabbit antibodies (green). Unbound free LLO is shown with green arrows. Scanned 20 images show absence of colocalization between MVs and LLO. Bar size: 10 µm; **(B)** MVs were negatively stained and unbound LLO labeled with gold particles is indicated with arrows. Twenty TEM images were analysed to see if MVs can bind and sequester LLO. Bar size, 200 nm; **(C)** ELISA, MVs are immobilized on the surface of microtiter plate coated with poly-D-lysine and purified LLO is added. ELISA shows LLO amount in each sample due to the binding either to poly-D-lysine or MVs. Examined samples are EGDeΔ*hly* MVs, LLO only, Δ*hly* MVs and LLO, or EGDe MVs and LLO. Error bars show SD from quadruplicates.
