# Supplementary figures and images for: A Novel Role of Listeria monocytogenes Membrane Vesicles in Inhibition of Autophagy and Cell Death
Source: Front Cell Infect Microbiol. 2017 May 3;7:154. doi: 10.3389/fcimb.2017.00154 (PMC5413512; doi:10.3389/fcimb.2017.00154)

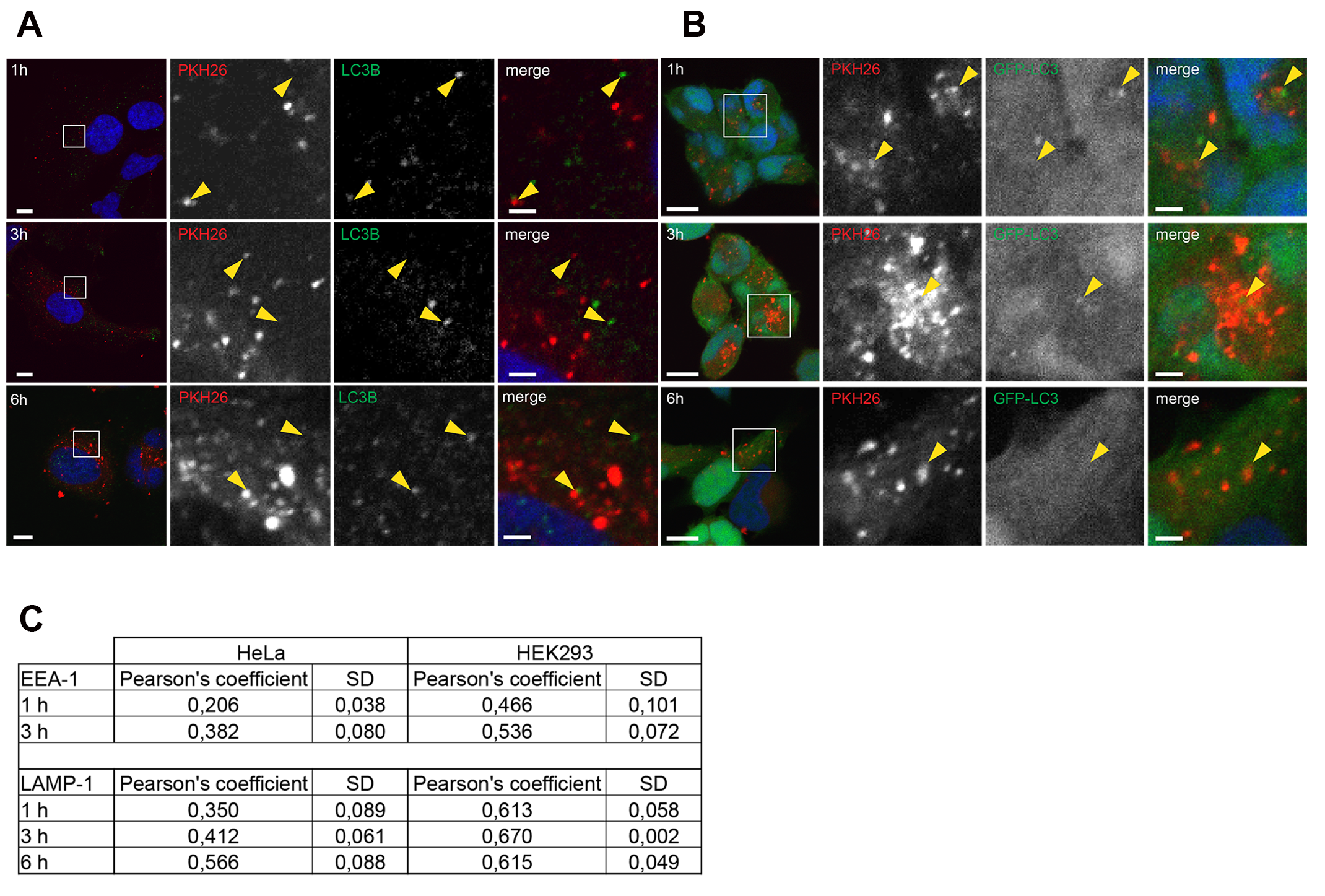

Supplement: Supplementary file 2 [file Image1.TIF]

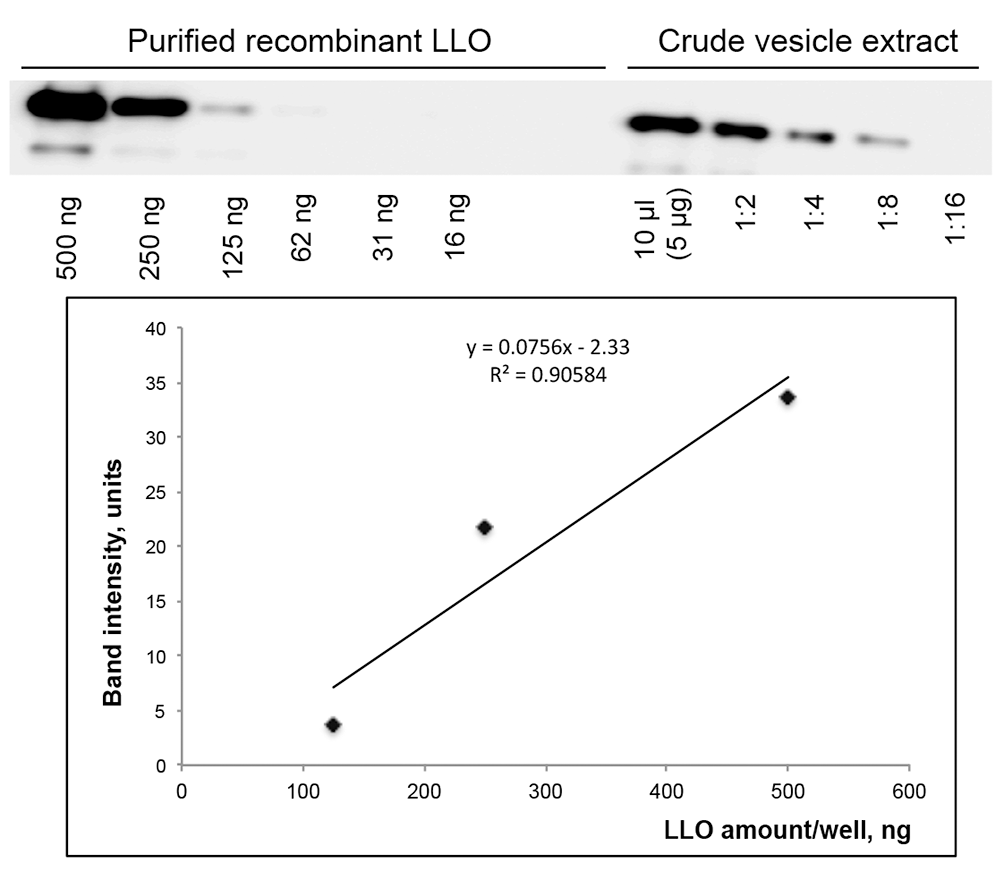

Supplement: Supplementary file 3 [file Image2.TIF]

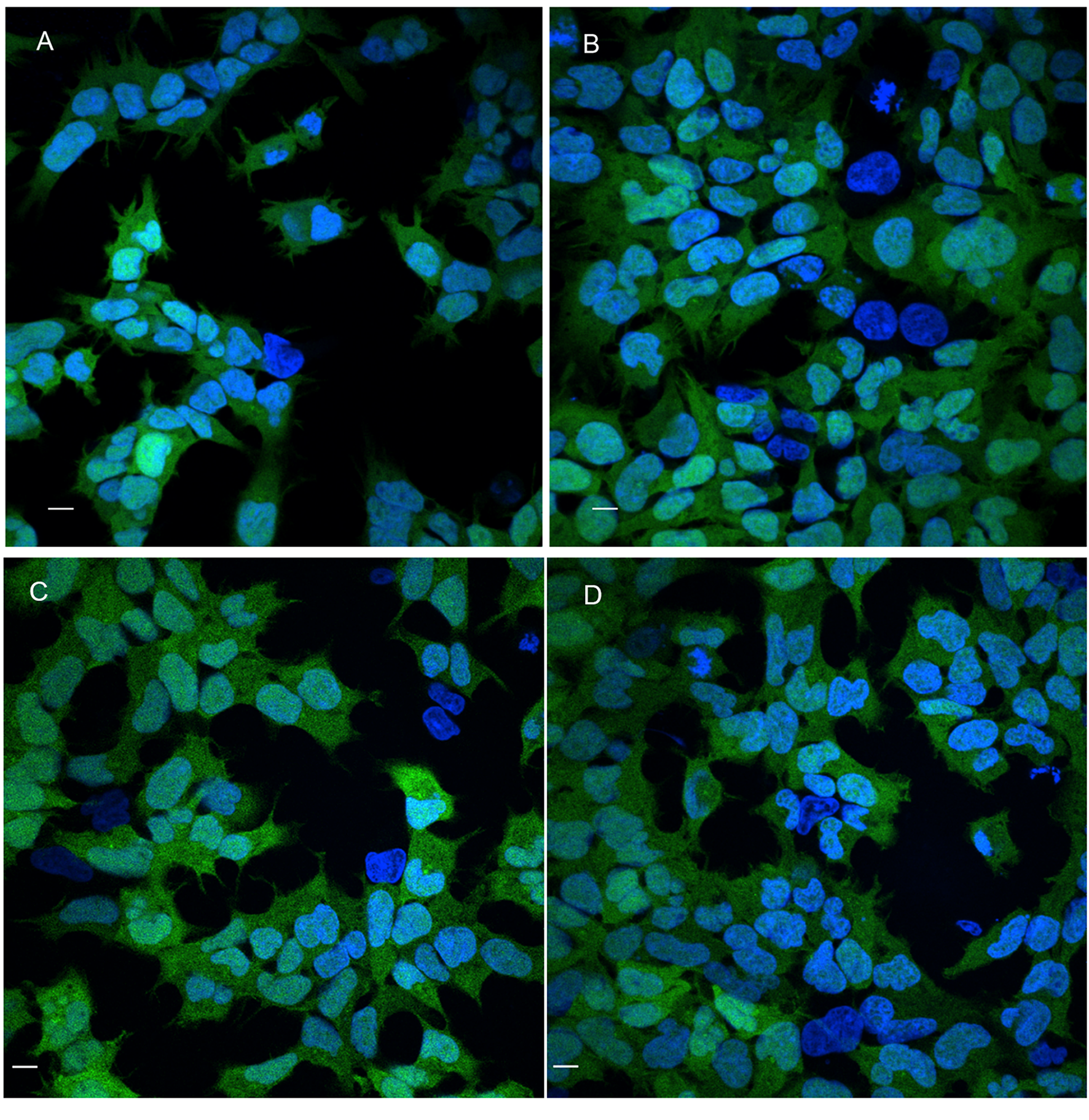

Supplement: Supplementary file 4 [file Image3.TIF]

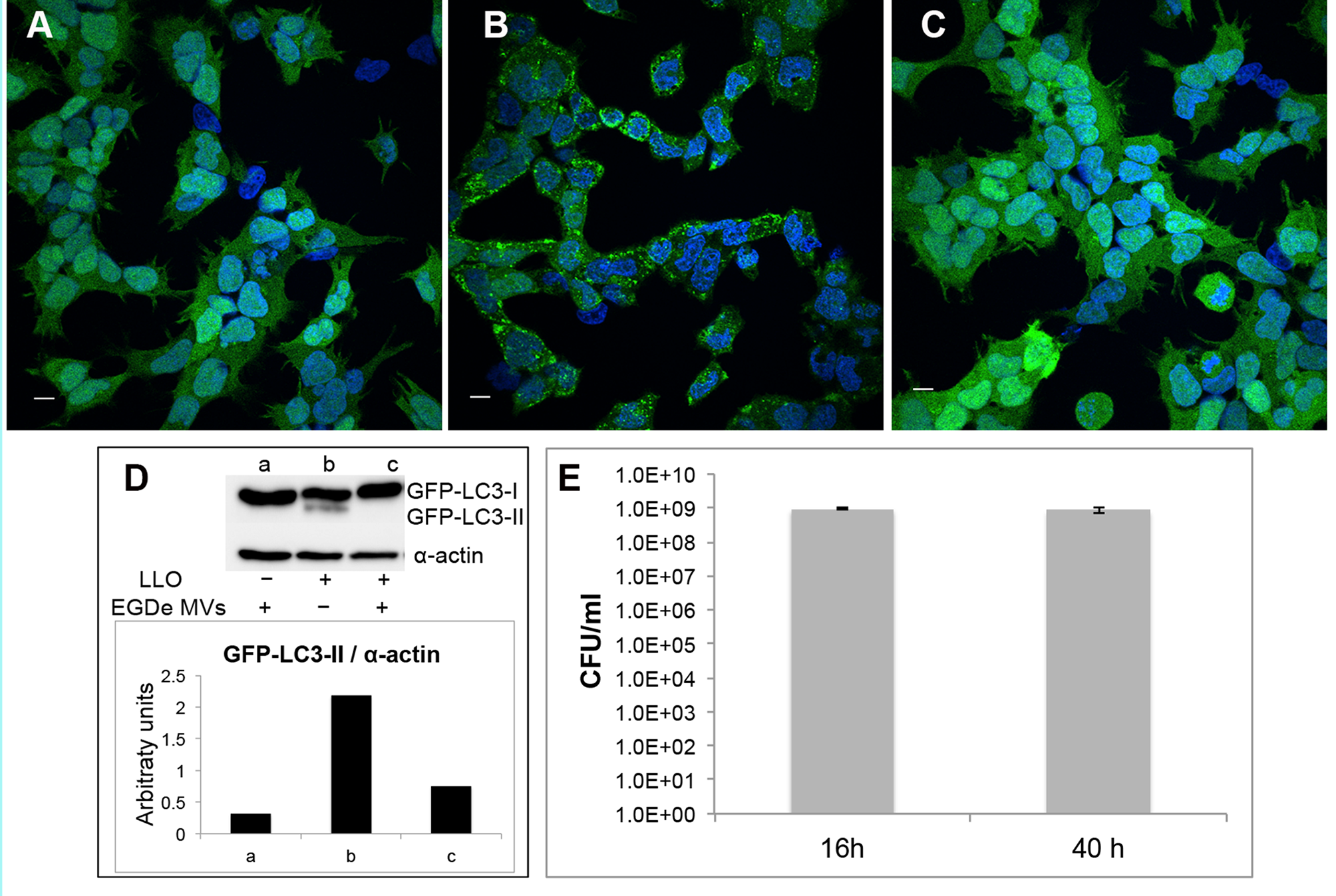

Supplement: Supplementary file 5 [file Image4.TIF]

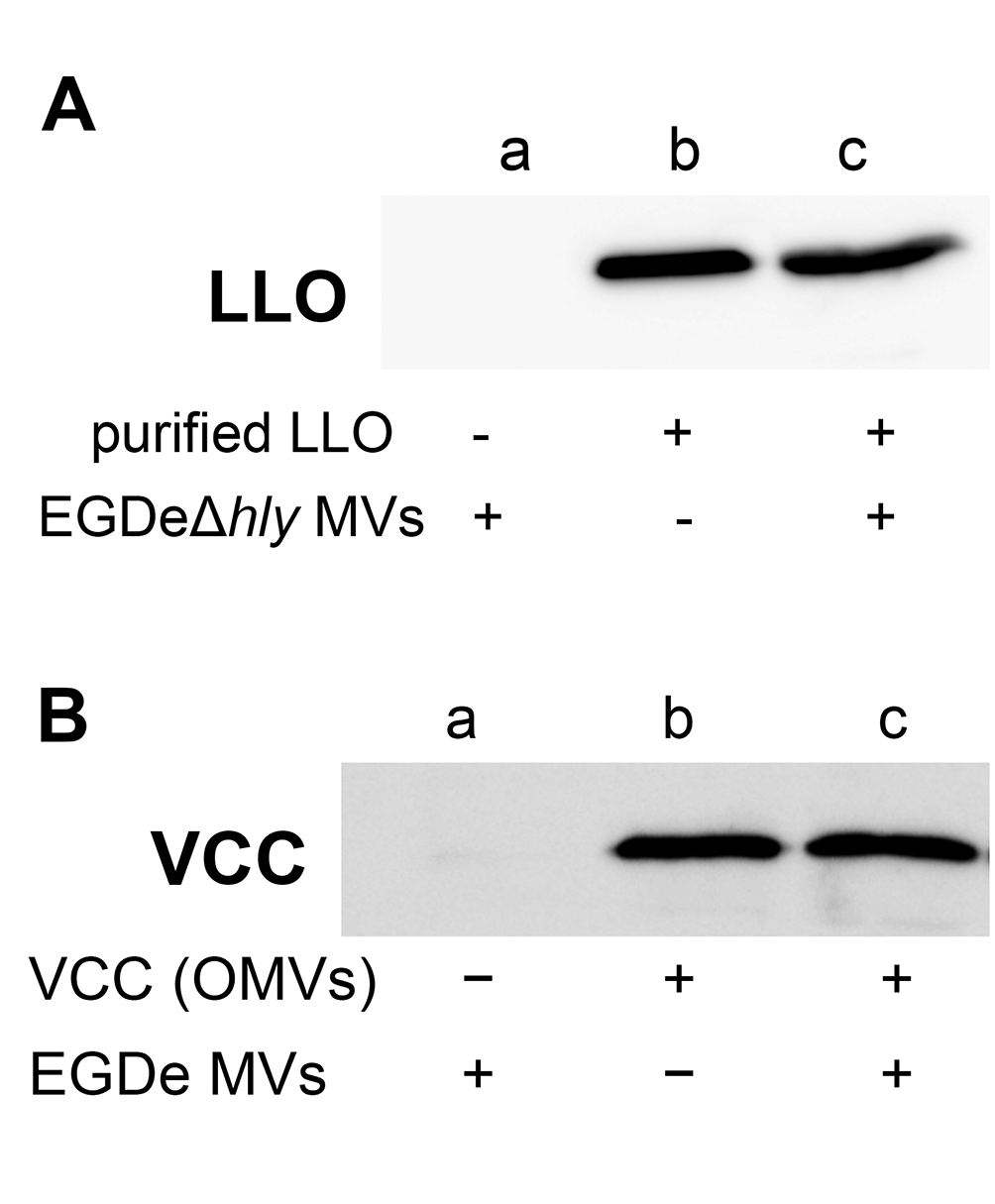

Supplement: Supplementary file 6 [file Image5.TIF]

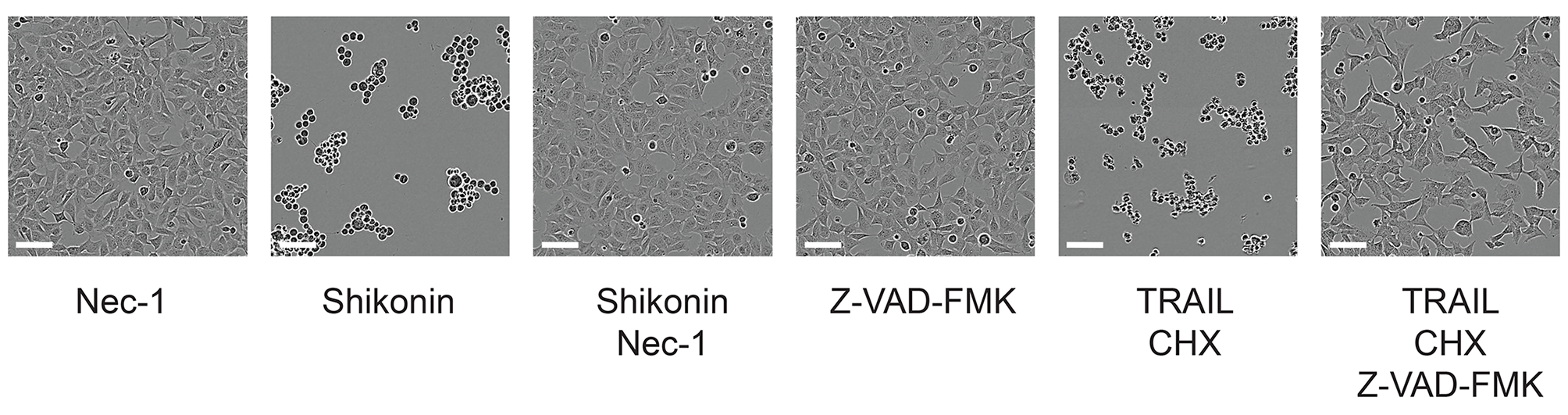

Supplement: Supplementary file 7 [file Image6.TIF]

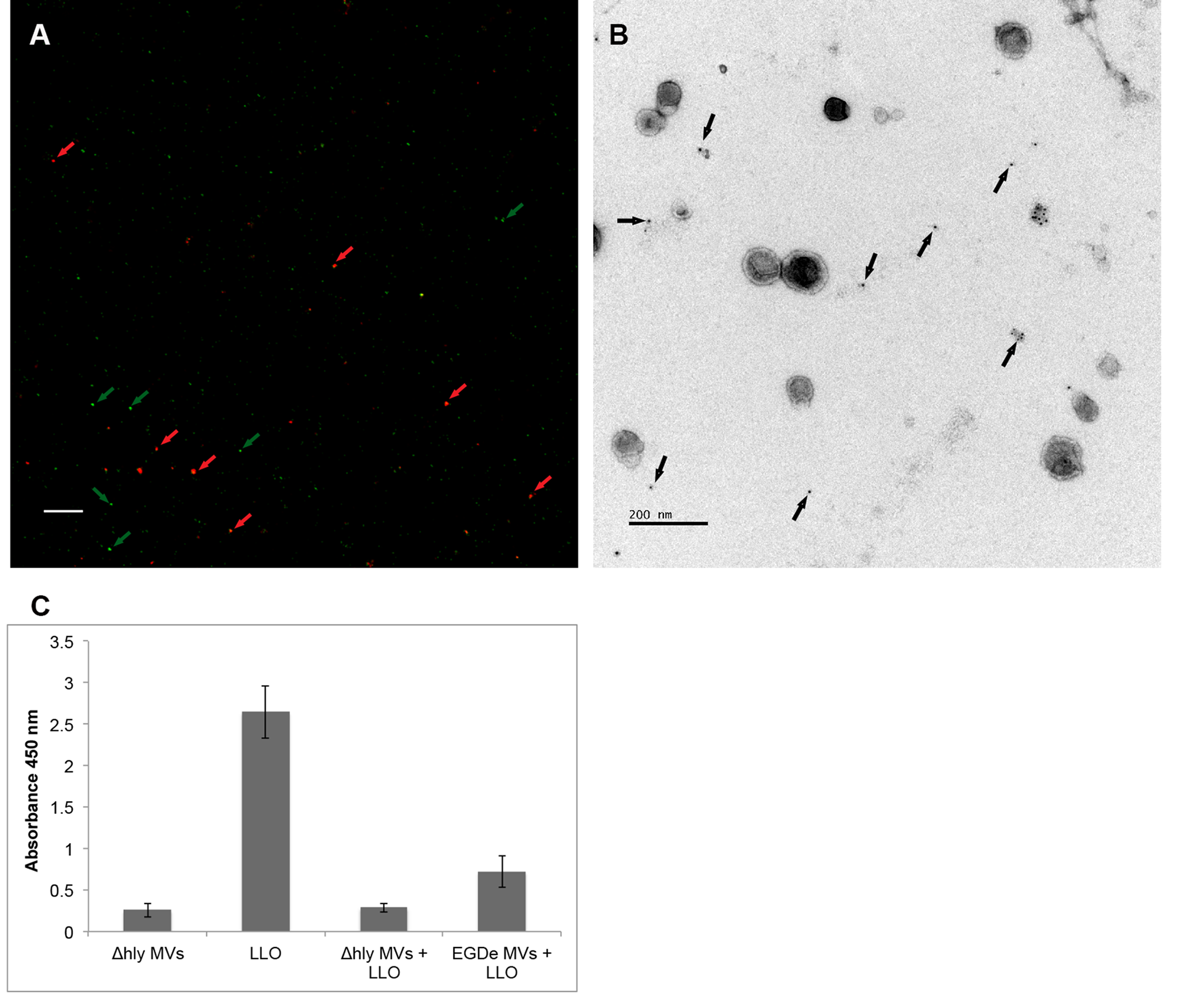

Supplement: Supplementary file 8 [file Image7.TIF]
